# Sidedness matters: single-cell perspectives on left- and right-sided colorectal cancer

**DOI:** 10.3389/fcell.2025.1720996

**Published:** 2025-12-10

**Authors:** Maria Yanova, Diana Maltseva, Alexander Tonevitsky

**Affiliations:** 1 Faculty of Biology and Biotechnology, National Research University Higher School of Economics, Moscow, Russia; 2 Shemyakin-Ovchinnikov Institute of Bioorganic Chemistry, Russian Academy of Sciences, Moscow, Russia

**Keywords:** colorectal cancer, single-cell RNA sequencing, left-sided and right-sided colorectal cancer, cell-cell interactions, cancer-associated fibroblasts, tumor-associated macrophages, immunotherapy, metastasis

## Abstract

Colorectal cancer (CRC) remains one of the leading causes of cancer-related morbidity and mortality worldwide. Tumor sidedness, distinguishing left- (LCRC) and right-sided (RCRC) cancers, has emerged as a critical clinical determinant, influencing patient prognosis and therapeutic response. However, the cellular and molecular mechanisms underlying these differences remain poorly understood. Recent advances in single-cell RNA sequencing (sc-seq) provide high-resolution insights into CRC heterogeneity, revealing distinct tumor, immune, and stromal cell populations and their context-specific interactions. In this review, we synthesize sc-seq studies that dissect the molecular programs driving progression, therapy resistance, and metastasis in CRC. We highlight malignant subclusters characterized by metabolic reprogramming and spatially organized oncogenic signaling; specialized immune cell states, including macrophage subsets, exhausted T cells, and mast cells, that shape tumor immunity; and stromal elements such as cancer-associated fibroblasts and endothelial tip cells that remodel the extracellular matrix, promote angiogenesis, and foster immune evasion. Importantly, sc-seq demonstrates that LCRC and RCRC represent distinct multicellular ecosystems with differential immune recruitment and stromal signaling, underscoring the need for sidedness-informed therapeutic strategies. We propose that future interventions should target cell-cell communication networks and spatially defined tumor–microenvironment interactions to overcome heterogeneity and improve clinical outcomes.

## 1 Introduction

Colorectal cancer (CRC) ranks as the third most common and second most fatal cancer globally, with approximately 1.9 million new cases and 905,000 deaths recorded in 2022 (Bray et al., 2024). At diagnosis, one-fifth of newly diagnosed patients present with metastatic CRC (mCRC) (Biller and Schrag, 2021). Moreover, up to 50% of patients initially diagnosed with localized CRC later develop metastases (Ciardiello et al., 2022). While the prognosis for mCRC has improved due to new treatment methods and the diagnostic process, the five-year survival rate remains critically low (under 13%) (Siegel et al., 2025). This creates a pressing clinical need to identify biomarkers and therapeutic targets to improve the effectiveness of treatment and predict the course of the disease.

This search is significantly complicated by pronounced tissue heterogeneity in CRC. Indeed, CRC is known to be highly heterogeneous in both primary tumors and metastatic lesions, which arises from multiple sources (Blank et al., 2018). Clinical studies have revealed significant differences in the incidence, outcomes, molecular and genetic profiles between anatomically distinct left-sided and right-sided CRC (LCRC and RCRC), first described by Bufill (1990). Although there is no clear distinction on the classification of CRC into LCRC and RCRC, the common definition is that RCRC refers to cancer proximal to the splenic flexure, whereas LCRC refers to cancer distal to the splenic flexure. This cutoff point is widely applied due to the embryonic development of the distal one-third from the hindgut, and the two-thirds of the transverse colon from the midgut. Additionally, the vascular supply is used to mark the embryologic origin with the superior mesenteric arteries supplying the midgut and the inferior mesenteric arteries supplying the hindgut (Ahmad Zawawi and Musa, 2022). Moreover, in some studies rectal carcinomas are classified together with LCRC due to similar embryological origin (Chen et al., 2021). Notably, despite the absence of defined molecular markers identifying phenotypically distinct CRC subtypes, classification by site of origin remains clinically useful - though not indicative of mutually exclusive signaling pathways (Cervantes et al., 2023). For example, left- or right-sided tumor location may help guide the choice between anti-EGFR and anti-VEGF therapy. It has been established that LCRC with unmutated RAS is more responsive to anti-EGFR treatment than RCRC (Cervantes et al., 2023). In contrast, for RCRC, anti-VEGF therapy is generally recommended regardless of pan-RAS status (Abdel Hamid et al., 2025). Consequently, tumor sidedness is now recognized as a critical prognostic and therapeutic determinant in CRC.

CRC heterogeneity manifests not only through genetic, transcriptional and translational alterations within cancer cells but also from the diverse cellular and extracellular matrix (ECM) composition of the tumor microenvironment (TME) (Blank et al., 2018). Single-cell analyses reveal that RCRC and LCRC are distinct multicellular ecosystems, characterized by pronounced immunologic and stromal differences (Liu B et al., 2024). These differences have direct therapeutic relevance, as the TME is increasingly recognized as a driver of tumor progression and a target for novel interventions (Li et al., 2022a). Biomarker development and treatment strategies should therefore integrate both tumor sidedness and intratumoral cellular heterogeneity to optimize clinical outcomes.

Bulk-tissue sequencing, one of the methods traditionally employed for consequent biomarker search and discovery, provides an overview of the cancerous tissue state as a whole, but fails to resolve tissue heterogeneity. In contrast, single-cell sequencing (sc-seq) allows to overcome this limitation by enabling the analysis of genomic or transcriptional data at individual cell resolution (Sun et al., 2025). This approach helps to point out the differences within the diverse cell populations comprising a tumor. Widely applied in the field of cancer research (e.g., colorectal, breast, brain, liver and lung cancers (Tang et al., 2019)), sc-seq provides critical insights into tumor heterogeneity and signaling pathways, including those promoting tumor progression and metastasis.

A key application of sc-seq is the analysis of intercellular signaling–a primary coordinator of cellular activities - predicted from gene expression data. In this context, the analysis of ligand-receptor pairs has become a useful technique: by detecting the coordinated expression of corresponding genes, researchers can characterize cellular communication and propose its potential influence on tumor growth and progression (Armingol et al., 2021). A typical workflow for such an analysis includes data acquisition, the construction of cell-cell communication networks, their computational inference, and, critically, experimental validation (Shao et al., 2020). Currently, several ligand/receptor tools exist to facilitate this process. The most common group of tools, including CellPhoneDB and CellChat, utilize curated ligand-receptor databases to assess co-expression across existing cell populations, employing permutation tests to identify significant interactions between cell types. The operational principles of current ligand-receptor analysis algorithms are reviewed in several publications (Armingol et al., 2021; 2024; Ma et al., 2021). Another group of tools includes NicheNet (Browaeys et al., 2020). Unlike methods that rely solely on ligand-receptor co-expression, NicheNet assumes that signaling to receiver cells causes downstream effects, such as changes in transcription factor activity. It therefore incorporates prior knowledge of signaling pathways to measure communication based on the enrichment of a receiver cell’s target genes. This approach effectively prioritizes cell-cell communication inferences based on downstream biological effects, thus helping to reduce the large number of interactions identified by other methods. However, this focus may also overlook genuine interactions that lack the expected downstream signature in the database.

Despite their utility, such computational methods have several limitations. A primary concern is their foundational assumption that transcriptomic data is a suitable proxy for cell-cell communication events. In reality, cells communicate via proteins (not RNA), and signaling is spatially constrained - neither of which can be fully captured by sc-seq. Furthermore, while these tools rely on curated ligand-receptor databases, true biological communication extends beyond the database-listed interactions. Consequently, the interactions they identify remain theoretical, making experimental validation essential. For these reasons, although these methods are widely applied in single-cell research due to their accessibility, their results must be interpreted with caution and confirmed experimentally.

This review synthesizes sc-seq findings in CRC to provide an overview of the molecular mechanisms underlying tumor therapy resistance, progression and metastasis in both overall CRC and the LCRC/RCLR subtypes, with an emphasis on context-specific interactions of cell populations inside tumors. Critically, sc-seq, in accordance with previous research, has identified that LCRC and RCRC are differing multicellular ecosystems, each defined by unique and varied immune and stromal signaling patterns, supporting the need for sidedness-informed therapeutic strategies. Consequently, further research into these spatially-defined intercellular communication networks and their subsequent therapeutic targeting holds promise for improving the clinical outcomes in CRC patients.

## 2 Prevalent cell types in colorectal cancer identified by single-cell sequencing

CRC is known to be comprised from a variety of cells participating in processes such as angiogenesis, tumorigenesis, immune evasion and metastasis (Fridman et al., 2020). Understanding the spatial and temporal distribution of these cellular components is therefore critical for developing precise therapeutic strategies. Sc-seq studies have recently enabled high-resolution mapping of CRC’s cellular landscape. The distribution of cells in overall and sided CRC is presented in Table 1 and will be discussed in more detail in the upcoming sections.

**TABLE 1:** Cellular heterogeneity in overall and sided CRC.

Cancer type	Tumor cells, % total	Immune cells, % total	Stromal cells, % total	Source
Overall CRC	24%–67%	T cells (8%–32%) B cells (3%–6%) Macrophages (7%)	Fibroblasts (7%–10%) Endothelial cells (2%–7%)	Lu et al. (2025), Qin et al. (2023), Wang et al. (2022)
LCRC	11%–30%	T cells (40%–65%) B cells (10%–20%) Myeloid cells (14%)	Fibroblasts (1.5%) Endothelial cells (0.7%–2%)	Hu et al. (2025), Liu D et al. (2024)
RCRC	20%–38%	T cells (31%–49%) B cells (13%–26%) Myeloid cells (13%)	Fibroblasts (2%) Endothelial cells (1%–2%)	Hu et al. (2025), Liu D et al. (2024)

### 2.1 Overall colorectal cancer cell composition

As summarized in Table 1, the TME of both overall CRC and sided subtypes (LCRC/RCRC) comprises heterogenous cell populations. These include epithelial cells, immune populations (T cells, B cells, monocytes/macrophages and mast cells) and stromal cells (cancer-associated fibroblasts (CAFs), endothelial cells and pericytes).

It should be noted that epithelial cells consistently dominate overall CRC tumors, although their abundance varies widely (24%–67%) (Lu et al., 2025; Qin et al., 2023; Wang et al., 2022). This variation likely reflects biological context (such as the tumor stage) and/or methodological differences in sample preparation and processing. Collectively, the analyzed studies demonstrate the diverse cellular composition of tumors and point to significant immune and stromal infiltration. Thus, a substantial fraction of the TME is composed of immune cells, with infiltrating T cells representing the predominant immune subset (8%–32% abundance). Stromal components, such as CAFs and endothelial cells, implicated in metastasis and therapy resistance are universally present, albeit at lower frequencies (typically <10% abundance) (Lu et al., 2025; Qin et al., 2023; Wang et al., 2022). Rare cell populations, such as mast cells and pericytes, are scarce but may exert a significant functional influence within the tumor.

Therefore, this cellular architecture of CRC highlights three potential therapeutic targets: 1) malignant epithelial populations, 2) immunomodulatory cell networks and 3) stromal mediators. Frequently, targeting a specific cell niche results in only partial, or even no, response to therapy (Fridman et al., 2020; Gallo et al., 2021; Leonard et al., 2021; Meyiah et al., 2025; Sphyris et al., 2021; Tauriello and Batlle, 2016). This limited efficacy may result from complex signaling pathways activated via cell-cell interactions. Sc-seq brings novel insights not only into cell-cell interactions, but also into the differences in such interactions across distinct anatomical locations. Consequently, targeting the newly identified pathways activated within these cellular components may represent a promising strategy to overcome therapy resistance and metastasis in CRC.

### 2.2 Left/right colorectal cancer cell composition

While LCRC and RCRC share the core cellular components of total CRC, their cell distribution differs (Table 1). Notably, RCRC demonstrates increased epithelial cell abundance in comparison with LCRC (20%–38% vs*.* 11%–30%). Such a difference may originate from their distinct embryonic development–while RCRC is midgut-derived, LCRC originates from the hindgut (Abdel Hamid et al., 2025). Interestingly, evidence from *Drosophila* models*,* which share structural and functional similarities with the mammalian colon and small intestine (Beumer and Clevers, 2021; Colombani and Andersen, 2020), has revealed that the midgut is characterized by a high rate of intestinal stem cell division and turnover, whereas the hindgut lacks constitutively active stem cells (Fox and Spradling, 2009; Micchelli and Perrimon, 2006). These findings may shed light onto the difference between TME in sided CRC.

Sidedness also shapes the immune landscape: according to sc-seq data RCRC demonstrates a slight B-cell enrichment (up to 26% vs. 20% in LCRC), while LCRC exhibits T cell dominance (up to 65% vs. 49% in RCRC). Interestingly, a recent study has revealed that patients with RCRC, but not LCRC have a more favorable clinical outcome with a higher density of tumoral B cells (Berntsson et al., 2016), suggesting distinct B-cell recruitment mechanisms. On the other hand, T cell populations can differentially influence CRC cell survival, as their effects are dictated by functionally distinct subsets; some are tumor-suppressive while others promote tumor progression (Guo et al., 2020). This suggests possible distinct molecular mechanisms for immune cell recruitment in the tumor.

The stromal components are present at similar levels in both subtypes (Table 1), constituting no more than 4% of the total cell population. Such a distribution points to their importance in the signaling of CRC tumors regardless of the anatomical site, although cellular differences and cell-cell interactions in stromal populations may be altered.

Collectively, the analyzed studies support the characterization of LCRC and RCRC as distinct multicellular ecosystems and suggest that left- and right-sided tumors employ different molecular mechanisms for immune cell recruitment.

## 3 Insights from single-cell sequencing in overall colorectal cancer

While the clinical significance of sidedness is of importance, single-cell technologies first aimed to clarify the profound heterogeneity inherent to all CRC tumors. Thus, this section of our review focuses on the foundational insights from sc-seq into the universal mechanisms of tumor therapy resistance, progression and metastasis in overall CRC, establishing the core cell types and corresponding interactions that are then differentially co-opted in left- and right-sided CRC tumors. Pathways specific to tumor sidedness will be discussed in subsequent sections.

### 3.1 Single-cell sequencing to elucidate tumor therapy resistance and progression in overall colorectal cancer

Despite advances in the treatment of CRC including chemotherapy, targeted therapy and immunotherapy, many patients exhibit either intrinsic or acquired resistance to tumor treatment. This ultimately leads to limited therapeutic efficacy and high rates of tumor recurrence. This challenge stems from the considerable diversity of resistance mechanisms and tumor progression pathways in CRC, which involve complex signaling interactions among tumor, immune, and stromal cells (Table 2). As the detailed study of these pathways is ongoing, sc-seq has provided insights into their function. The contribution of single-cell technologies will be detailed throughout this review.

**TABLE 2:** Cell-cell communication in colorectal cancer: predicted ligand-receptor interactions and their functional roles in overall and sided colorectal cancer.

Interacting cell types	Key ligand-receptor interactions	Ligand-receptor description	Potential function/consequence	Source
Overall CRC				
Tumor-stromal/stromal-tumor				
Nu^High^ cells - fibroblasts	PLA2G2A-A5B1 MIF-TNFRSF14 COL1A1/COLIA2-ITGB1	Phospholipase – integrin receptor Macrophage migration inhibitory factor – Tumor necrosis factor receptor Collagen – integrin receptor	Not indicated	Liu J et al. (2024)
Nu^High^ cells – endothelium	PLA2G2A-A5B1 MIF-TNFRSF10D MIF-TNFRSF14	Phospholipase – fibronectin receptor Macrophage migration inhibitory factor – Tumor necrosis factor receptor	Not indicated	Liu J et al. (2024)
pMMR tumor cells – *CXCL14* ^+^ fibroblasts	*IHH-PTCH1*	Indian hedgehog – patched receptor	Regulation of differentiation of *CXCL14* ^*+*^ fibroblasts and dysregulation of ECM organization to restrict immune cell tumor infiltration in pMMR.	Feng et al. (2024)
Tumor cells – tip cells	*VEGFA-NRP1/2*	Vascular endothelial growth factor – Neuropilin receptor	Promotion of angiogenesis and lymphatic sprouting	Xie et al. (2024)
Stromal cells – tumor cells	RPS19-C5AR1	Ribosomal protein – complement receptor	Promotion of tumor growth, angiogenesis, tissue remodeling and EMT	Xiao et al. (2024)
*CTHRC1* ^+^ fibroblasts – Tumor cells group	COL1A1-ITGB1 LAMA1-ITGA3 WNT5A-LRP6/FZD3 THBS1-ITGAV	Collagen – Integrin receptor Laminin – Integrin receptor Wnt – frizzled receptor Thrombospondin – Integrin receptor	Enhancement of tumor cell migration and invasion	Lu et al. (2025)
*FAP* ^+^ fibroblasts – *SPP1* ^+^ macrophages	COL1A1/LAMA1-ITGB1 WNT5A-FZD2 CCL3-CCR5 RARRES2-CMKLR1	Collagen/laminin – Integrin receptor Wnt – Frizzled receptor Chemokine – Chemokine receptor Chemerin – Chemerin receptor	Influence on cell adhesion properties, enhancement of recruitment and pro-inflammatory activity of *SPP1* ^+^ macrophages	Qi et al. (2022)
Tumor-immune/immune-tumor				
Nu^High^ cells – macrophages	PLA2G2A – A4B1 PLA2G2A – A5B1	Phospholipase – integrin receptor	Not indicated	Liu J et al. (2024)
Immune-immune				
*C1QC* ^+^ TAMs – T cells	CXCL10-CXCR3	Chemokine – chemokine receptor	Recruitment and activation of T cells	Zhang et al. (2020)
*LAMP3* ^*+*^ DCs - *CXCL13* ^+^ T cells in dMMR and PR/CR	PD-L1-PD1	Programmed cell death protein – Programmed cell death protein receptor	Not indicated	Feng et al. (2024)
LpMs – T cells	CD28^−^CD80 CLTA4-CD80 CD28-CD86 CLTA4-CD86	Cluster of differentiation – cluster of differentiation coreceptor	Regulation of T cell response in the colonic mucosa	Domanska et al. (2022)
Immune-stromal/stromal-immune				
*SPP1* ^*+*^ TAMs - fibroblasts	SDC2-MMP2	Syndecan receptor - Matrix metalloproteinase	Tumor growth and metastasis	Zhang et al. (2020)
Stromal cells (epithelial cells) - LpMs	LGALS9-HAVCR2 HLA-G-LILRB2 HLA-G-LILRB1 TNFSF10-RIPK1 GAS6/PROS1-AXL GAS6-MERTK CD47-SIRPA CD52-SIGLEC10	Galectin - Hepatitis A virus cellular receptor Human leukocyte antigen - Immunomodulatory receptor Tumor necrosis factor-related apoptosis-inducing ligand – protein kinase interacting receptor Growth arrest specific ligand/protein S – tyrosine kinase receptor Growth arrest specific ligand – tyrosine kinase receptor Cluster of differentiation - signal regulatory protein Cluster of differentiation - sialic acid-binding Ig-like lectin	Macrophage activation, M2-like polarization and “do not eat me” signals secretion	Domanska et al. (2022)
Fibroblasts and endothelial cells – mast cells	KITLG-KIT	Stem cell factor – receptor tyrosine kinase	Promotion of MC activation	Xie et al. (2023)
LCRC				
Tumor-stromal/stromal-tumor				
Tumor cells - fibroblasts	PLAU-PLAUR	Urokinase plasminogen activator - urokinase receptor	Formation of inflammatory CAFs	Hu et al. (2025)
Fibroblasts- tumor cells	Collagens-a1b1 TGFB1-TGFBR1	Collagen – integrin receptor Tissue growth factor – tissue growth factor receptor	Tumor cell growth and proliferation	W. Guo et al. (2022)
Endothelial cells – tumor cells	COL4A1/COL4A2-a1b1	Collagen – integrin receptor	Not indicated	Liu J et al. (2024)
Tumor-immune/immune-tumor				
M1/M2 macrophages – tumor cells	TNF-RIPK1/TNFRSF1B TNFSF10-VSIR/RIPK1/TNFRSF1B	Tumor necrosis factor – protein kinase interacting receptor/tumor necrosis factor receptor Tumor necrosis factor – immunomodulatory receptor/protein kinase interacting receptor/tumor necrosis factor receptor	Not indicated	Ji et al. (2024)
Tumor cells – B cells	CCL28-CCR10	Chemokine – chemokine receptor	Immunosuppressive signaling	Hu et al. (2025)
PS^high^ Tumor cells – *PLTP* ^*+*^ Macrophages	CSF1-CSF1R	Colony stimulating factor – colony stimulating factor receptor	Maintenance of *PLTP* ^*+*^ macrophage anti-inflammatory phenotype	Liu B et al. (2024)
Immune-immune				
B cells – Myeloid cells	CD52-SIGLEC10	Cluster of differentiation - sialic acid-binding Ig-like lectin	Inhibition of proliferation and activation of immune cells	Ji et al. (2024)
Stromal-immune/immune-stromal				
*MYH11* ^+^ fibroblasts – myeloid cells	CD74-MIF SPP1-CD44	Cluster of differentiation - macrophage migration inhibitory factor Osteopontin – cluster of differentiation	Not indicated	C. Wang et al. (2024)
RCRC				
Tumor-stromal				
Tumor cells - fibroblasts	TIMP2-MMP2	*Tissue inhibitor of metalloproteinases – Matrix metalloproteinase*	Promotion of angiogenesis and ECM degradation	Hu et al. (2025)
Tumor cells – endothelial cells	DCN-VEGFR2 SEMA3B-NRP2/NRP1	Decorin – vascular endothelial growth factor receptor Semaphorin – Neuropilin receptor	Promotion of tumor invasion, metastasis and the increase in vascular permeability and cell survival	Hu et al. (2025)
Tumor-immune/immune-tumor				
IS^high^ tumor cells-*SPP1* ^+^ macrophages	CCL15-CCR1 PDL1-CD80	Chemokine – chemokine receptor Programmed cell death protein – cluster of differentiation	Recruitment of *SPP1* ^*+*^ macrophages and the decrease in the efficiency of antigen presentation and activation of T cells	Liu B et al. (2024)
IS^high^ tumor cells-*CD161* ^+^ CD8^+^ innate-like T cells	CXCL16-CXCR6	Chemokine – chemokine receptor	Recruitment of *CD161* ^+^ CD8^+^ innate-like T cells and interaction with PD-L1	Liu B et al. (2024)
Macrophages – tumor cells	FPR1-SAA1	Serum amyloid - formyl peptide receptor	Promotion of the production of cytokines/chemokines	Hu et al. (2025)
Trm, Tex CD8^+^ cells, Treg, Tfh CD4^+^ cells– tumor cells	TIGIT-NECTIN2 SEMA4D-PLXNB2 CD8A−CEACAM5 ADGRE5−CD55	T cell immunoreceptor – nectin receptor Cluster of differentiation – plexin receptor Cluster of differentiation – cell adhesion molecule Cluster of differentiation – cluster of differentiation coreceptor	Not indicated	Liu D et al. (2024)
Immune-immune				
*SPP1* ^+^ macrophages-*CD161* ^*+*^ CD8^+^ innate like T cells	SPP1-CD44	Osteopontin – cluster of differentiation	The dampening of cytotoxic T cell capability	Liu B et al. (2024)
*LUCAT1* ^+^ monocytes – monocytes and macrophages	CD44−CD74	Cluster of differentiation – cluster of differentiation coreceptor	Not indicated	Shang et al. (2024)
Monocytes – Monocytes and Neutrophils	Reduced ANXA1-FPR1	Annexin – Formyl peptide receptor 1	Promotion of immune evasion and enhancement of immunosuppressive functions of Tregs	Shang et al. (2024)
B cells – Myeloid cells	SEMA4A-PLXND1	Semaphorin – Plexin receptor	Promotion of macrophage migration	Ji et al. (2024)
Immune-stromal/stromal-immune				
M1 macrophages – endothelial cells	TGFB1-TGFBR1	Tissue growth factor – tissue growth factor receptor	Not indicated	W. Guo et al. (2022)

#### 3.1.1 Mechanisms of tumor cell signaling leading to colorectal cancer progression

The progression of CRC is driven not only by the intrinsic properties of tumor cells, but also involves their dynamic interactions with the TME. The tumor mass itself is a complex ecosystem, primarily composed of tumor cells, with stromal cells providing essential support (Liu and Dilger, 2025). Immune cells within this niche add another layer of complexity, exerting opposing influences on tumor progression through pro- and anti-tumor mechanisms. While foundational therapies like surgery, radiotherapy, and chemotherapy directly target tumor cells, the disruption of the TME has become a major therapeutic focus. The pursuit of more effective treatments is now being guided by insights from sc-seq and spatial transcriptomic studies, which have begun to unravel these complex mechanisms (Table 3).

**TABLE 3:** Transcriptomic features of tumor cells in overall and left- and right-sided CRC progression revealed by single-cell sequencing and spatial transcriptomics.

Key findings	Source
Overall CRC	
- Upregulation of PPAR signaling pathway-associated genes when compared with normal cells - PPAR signaling inhibition through PPARγ inhibitor (GW9662) suppressed growth and accelerated apoptosis of tumor epithelial cells in patient-derived tumor organoids (as effective as 5-FU treatment at 30 uM concentrations)	R. Wang et al. (2022)
- Bulk TCGA analysis identified MTRGs DEGs between normal and tumorous tissues to build a four-gene prognostic model - Only *TIMP1* gene expression level significantly increased during epithelial cell pseudo-time analysis (HPA database confirmed high TIMP1 expression in CRC) - High-risk patients demonstrated lower IC50 levels in response to elesclomol, shikonin, and bryostatin-1 (agents predicted to target TIMP1)	Li et al. (2022b)
- Tumor cell subclusters enriched with gene response to HDAC inhibitors (*ATF3* and *CAV1*), inflammatory genes (*LXN* and *PGM1*) and tumor metastasis-related hypoxia (*WSB1*) were identified - Tumor cells expressed *TMSB4X* at high level (a validated therapeutic target) - Subregions of the tumor included enhanced focal adhesion dynamics, metabolic and hypoxia-response modules, suggesting their involvement in tumor progression	Xiao et al. (2024)
- Elevated nucleotide metabolism in comparison with normal epithelial cells - Elevated EGFR, hypoxia, MAPK and TGF-β signaling in tumor regions - The hub gene for nucleotide metabolism *NME1* (identified through sc-seq) suppresses migration and stemness *in vitro* and predicts immunotherapy response in CRC patients	Liu J et al. (2024)
LCRC	
- LCRC-exclusive group of cancer cells is enriched for genes (*TFF1, TFF2, AGR3, MUC5AC* etc.) involved in mucosal protection/epithelial healing. Higher expression of these genes was IHC-verified in LCRC vs*.* RCRC. - This group demonstrated upregulated estrogen, ERBB, TNF etc. signaling pathways, enhanced cell death (apoptosis, autophagy) and metabolism (lipid, amino acid and oxidative phosphorylation)	W. Guo et al. (2022)
- *MMP7* ^+^ cancer cells appear specifically in advanced LCRC samples, positively express “extracellular matrix remodeling” gene panel and have significantly higher metastatic potential compared with other cancer subclusters	Hu et al. (2025)
- Lower malignance score in LCRC vs*.* RCRC cancer cells - *LPCAT2* ^+^ tumor cells have a lower malignancy score than *LPCAT2* ^-^ population and are significantly increased in the inflammatory phase of colitis-associated colon cancer mouse model; a significantly higher proportion of *LPCAT2* ^+^ cells in LCRC vs*.* RCRC (confirmed by IHC in tumor tissues) - *LPCAT2* overexpression/downregulation significantly inhibits/enhances cell proliferation and colony-formation ability *in vitro*, as well as repress/stimulate tumor growth and proliferation marker ki67 *in vivo* - *LPCAT2* acts as a ferroptosis inducer via the *PRMT1/SLC7A11* axis, suppressing CRC cell proliferation	Cao et al. (2024)
- Proliferation, stemness and immune secretory meta-programs show inverted gene expression patterns in malignant epithelium from LCRC and RCRC tumors (validated in several cohorts) - The PS meta-program is characterized by expression of genes associated with proliferation (*UBE2C* and *MYBL2*) and stemness-related genes (such as *ALDH1A1*), predominantly expressed in LCRC. - GSEA revealed that glycolysis- and proliferation-related pathways are significantly enriched in PS^high^ cancer cells (verified by IHC staining of LDHA and ki67) - PS^high^ malignant epithelial cells are enriched in high glycolytic cell areas, colocalizing with *PLTP* ^*+*^ TAMs, activated Tregs, and *LAYN* ^+^ CD8^+^ T cells	Liu B et al. (2024)
- Tumor cells in LCRC exhibit minimal expression of MHC I molecules. Patients with lower levels of MHC I expression have a worse prognosis in comparison with those with higher levels	Liu D et al. (2024)
- Epithelial cells in LCRC show elevated *PHLDA2*, a gene linked to lymph node metastasis	C. Wang et al. (2024)
RCRC	
- GSVA analysis of cancer cells shows that several metabolic and inflammatory related hallmarks are significantly enriched in MAC, especially hallmark-glycolysis (increases in a gradient among AC, pMAC, and MAC)	Xu et al. (2025)
- Increased functional state of hypoxia compared to LCRC: high expression of *HIF1A* according to sc-seq (verified additionally in TCGA and cancer tissue). Pathways “metabolism” in *LYZ* ^+^ cancer cells, “stress” in *TFF1* ^+^ cancer cells and chemokines for *MUC5AC* ^+^ cancer cells are activated in response to hypoxia in RCRC.	Hu et al. (2025)
- Proliferation, stemness and immune secretory meta-programs show inverted expression patterns in malignant epithelium from LCRC and RCRC tumors (validated in several cohorts) - The IS meta-program has increased expression of MHCII molecules (*CD74*, *HLA-DRB1*, and *HLA-DPA1*) and secretory protein molecules (*REG4*, *AGR2*, and *AGR3*), with *AGR2* and *REG4* specifically expressed in RCRC. - IS^high^ malignant epithelium is in close proximity to the hypoxic tumor core and is collocated with *SPP1* ^+^ TAMs and *CD161* ^+^ CD8^+^ T cells	Liu B et al. (2024)
- Tumor cells in RCRC exhibit a high expression of MHC I molecules. In patients with colon cancer, those with lower levels of MHC I expression experienced a significantly worse prognosis compared to those with higher levels	Liu D et al. (2024)

An example of a therapeutically targetable pathway intrinsic to tumor cells is the peroxisome proliferator-activated receptor (PPAR) signaling pathway. Wang et al. demonstrated marked upregulation of the PPAR signaling in tumor epithelial cells relative to normal tissue (Wang et al., 2022). Functionally, inhibition of this pathway suppressed growth and activated apoptosis in patient-derived CRC organoids - an effect comparable to the standard chemotherapy drug 5-fluorouracil (5-FU). This suggests PPAR is a promising therapeutic target for CRC tumor cells, particularly relevant given that, despite substantial preclinical evidence, PPAR inhibitors have not yet seen widespread effective use in CRC patient treatment. Beyond obtaining data in support of well-studied pathways, new signaling networks have been described with the help of single-cell transcriptomic technologies. One such network was identified by utilizing bulk RNA sequencing and sc-seq findings to build a four-gene prognostic model using membrane-tension related genes (MTRGs) in patients from the TCGA cohort (Li et al., 2022b). Among them, the expression of tissue inhibitor of metalloproteinase 1 (*TIMP1*) notably increased during epithelial pseudotime trajectory analysis, potentially linking it to CRC progression. Furthermore, high-risk TCGA patients showed increased sensitivity (lower IC50) to the drugs elesclomol, shikonin, and bryostatin-1 - agents predicted to have high affinity for TIMP1. Given that previous studies have implicated *TIMP1* in promoting tumor proliferative abilities and metastasis both *in vitro* and *in vivo* (B. Ma et al., 2022; Rao et al., 2022; Song et al., 2016), the findings position the TIMP1-centered signaling axis as a potential therapeutic target in CRC.

The use of single-cell technologies extends beyond identifying single potential therapeutic targets/signaling pathways - they allow for the identification of functional heterogeneity within the tumor cell population itself. As opposed to being a uniform mass, CRC tumors contain distinct subclusters of malignant cells characterized by differing molecular programs driving tumor progression. For instance, sc-seq has uncovered malignant cell subclusters enriched in genes associated with histone deacetylase (HDAC) inhibitor response (*ATF3*, *CAV1*), inflammation (*LXN*, *PGM1*), and hypoxia-linked tumor metastasis (*WSB1*) (Xiao et al., 2024), highlighting their importance in tumor progression. This cellular diversity, which allows the tumor to adapt and resist therapy, is further organized in a specific spatial context. Spatial transcriptomics of CRC tumor cryosections revealed high expression of thymosin beta-4 (*TMSB4X*) in tumor cells - a protein involved in actin binding, cell migration, and inflammation (Gemoll et al., 2015; Nemolato et al., 2012). Tumor subregions further exhibited upregulated pathways related to focal adhesion dynamics, ECM, metabolism, and hypoxia response, implicating these processes in tumor progression. This spatial organization is additionally characterized by active signaling crosstalk, such as the crosstalk between stromal and tumor regions via the tumor progression and immune evasion (C5AR1-RPS19) signaling axis. These findings demonstrate that CRC progression is driven by functional heterogeneity among malignant cells, their spatially organized molecular gene expression upregulation (*TMSB4X* in tumor cells), regional activation of key oncogenic pathways and active signaling crosstalk between tumor and stromal compartments.

Metabolic reprogramming, specifically regarding nucleotide synthesis, was identified by single-cell analysis as a driver of CRC progression, with elevated levels detected in tumor versus normal tissue (Liu J et al., 2024). Tumor cells exhibiting high nucleotide metabolism (termed NU^high^ cells) showed extensive communication with stromal and immune cells, acting as coordinators of the tumor-stromal-immune interactions (Table 2). Interestingly, NU^high^ cells signal via macrophage migration inhibitory factor (*MIF*) and secreted phospholipase A2 (*PLA2*) to engage TNF receptors/integrins on stromal/immune cells, whereas NU^high^ cells receive signals from fibroblast- and macrophage-secreted B cell-activating factor (*BAFF*) through the transferrin receptor (*TFRC*) (Liu J et al., 2024). Spatial transcriptomics, in turn, confirmed elevated nucleotide metabolism in CRC tumors alongside increased EGFR, hypoxia, MAPK, and TGF-β signaling. Importantly, the nucleoside diphosphate kinase A *(NME1)* gene was identified as a key regulator of nucleotide metabolism. Functional validation of such findings in CRC xenograft models demonstrated that *NME1* inhibits migration and reduces stemness, confirming its importance in tumor biology. Thus, nucleotide metabolism is not only a metabolic hallmark of CRC but acts as a central focal point that coordinates tumor-stromal-immune signaling (via MIF/PLA2/BAFF/TFRC) and spatially co-localizes with oncogenic pathways. The identification of *NME1* as a key regulator whose activity suppresses migration and stemness highlights nucleotide metabolism (and *NME1* specifically) as a therapeutically relevant pathway in CRC.

In total, overall CRC progression is driven by dysregulated signaling within tumor cells that combine both intrinsic oncogenic pathways and the intercellular signaling within the TME. The newly identified important mechanisms include, firstly, metabolic reprogramming with elevated nucleotide metabolism (via *NME1*) that allows the communication of tumor-stromal-immune cells through both outgoing (MIF-PLA2) and incoming (BAFF-TFRC) signals, all while being spatially colocalized with EGFR/hypoxia/MAPK/TGF-β pathways. Secondly, subregions within the tumor demonstrate spatially organized signaling through PPAR, focal adhesion/ECM remodeling and hypoxia-driven metastasis. Lastly, the novel mechanisms include functional heterogeneity where malignant subclusters in CRC tumors drive progression via HDAC inhibitor-responsive genes (*ATF3/CAV1*), inflammatory mediators (*LXN/PGM1, TMSB4X*) and the stromal-tumor crosstalk for tumor progression and immune evasion (C5AR1-RPS19 axis). Targeting the mentioned pathways, in particular PPAR, TIMP1, and NME1-regulated pathways, serves as a promising strategy for CRC patient treatment.

#### 3.1.2 Mechanisms of immune cell signaling in CRC progression

The immune landscape within CRC is a complex network of specialized cells whose functions are defined by their identity, location within the tumor and cellular communication networks. Single-cell technologies are helping to navigate through this complexity, revealing how specific immune subsets influence progression and helping to guide the direction for novel therapeutic strategies development (findings summarized in Table 4).

**TABLE 4:** Transcriptomic features of immune cells in overall and sided CRC progression revealed by single-cell sequencing and spatial transcriptomics.

Cell type	Key findings	Source
Overall CRC		
Macrophages	- *C1QC* ^+^ TAMs express genes related to phagocytosis and antigen presentation - *SPP1* ^+^ TAMs are enriched for regulators of angiogenesis and metastatic liver cancer pathways and are increased in tumor vs*.* normal mucosa - Anti-CSF1R treatment reduces overall TAM numbers in Renca tumor-bearing mice but reveals a resistant population with decreased F4/80 expression. Resistant TAMs preferentially express angiogenesis (*VEGFA*) and immunosuppression (*CD274, ARG1*) genes and are associated with tumor vascularization	Zhang et al. (2020)
	- LpM are identified to be proinflammatory and antigen-presenting/phagocytic (the latter positioned in the subepithelial region). They express proinflammatory genes (e.g., *S100A9, CXCL9*) and genes involved in antigen presentation (e.g., *HLA-DRA*) - *LYVE1* ^+^ SmMs display a transcriptomic profile characterized by low antigen-presenting capacity but high chemotactic and tissue-protective properties - MMs are dominated by clusters expressing genes associated with homeostatic functions (e.g., *C1QC, COLEC12, LYVE1, CCL3*)	Domanska et al. (2022)
	- Upregulated genes in TAMs include receptors bearing ITIM (such as *SIRPA*, *CLEC4A*, *SIGLEC9* and *LILRA/Bs*). Their expression levels are elevated in tumor tissues compared to paracancerous tissues and normal tissues in patients with stage II-IV of the disease - *SIRPA* ^−/−^ mice develop smaller tumors in subcutaneous MC38 model and have longer overall survival. Rechallenged tumors are completely rejected. Flow cytometry analysis demonstrates an increase in the number of macrophages in *SIRPA* ^−/−^ mice, in the expression levels of MHC-II and in CD8^+^ T cells - There is a reduction in the number of tumors and significantly smaller tumors in the intestines of *SIRPA* ^−/−^ mice than in those of WT mice. Histological analysis revealed that chronic inflammation in the colon of AOM/DSS-treated *SIRPA* ^−/−^ mice is significantly attenuated compared to WT mice - Sirpα KO macrophages facilitate T cell recruitment into tumors via Syk/Btk-dependent Ccl8 secretion	Huang et al. (2024)
T cells	- CD8^+^ T cells: Tem phenotype is associated with a less dysfunctional transcriptomic state and positive clinical outcomes. *GNLY* ^+^ *CD103* ^+^ T cells are found within tumors (absent in normal colon), demonstrate loss of cytokine production, and do not correlate with positive outcomes in CRC. - CD4^+^ T cells: cytotoxic CD4^+^ T cell subset expresses high levels of effector molecules and coinhibitory molecules (e.g., PD-1) - Tregs: *Helios* ^+^ Tregs are associated with improved patient outcomes and are present in early-stage cancer. pTregs are associated with poor outcomes (stage-independent), express high levels of *CD38* and *LAG3*, and produce elevated IL-10, suggesting strong immunosuppressive activity	Masuda et al. (2022)
Mast cells	- The proportion of activated vs*.* resting MCs significantly change in CRC (validated across TCGA and 9 additional cohorts) - DEGs enriched in CRC MCs (vs*.* normal) include *TMEM176B* and *CD52*, the latter a known marker for neoplastic MCs. DEG analysis (GO) revealed cell activation pathways as most enriched. CRC MCs also show higher expression of their key receptors and mediators - Activated MCs are associated with TNFα signaling via NF-kB and enriched in MHC I and II genes, whereas resting MCs are associated with an angiogenesis related pathway (GSVA analysis) - High MC signature is associated with favorable outcome in TCGA patient datasets (high expression of five MC signature genes is associated with better disease-free survival) - *In vitro* experiments demonstrate that KITLG-activated P815 cells show a decrease in cell proliferation and migration - the KITLG/KIT signaling pathway may be an important mechanism for CRC progression inhibition	Xie et al. (2023)
pMMR/dMMR cells	- pMMR tumors exhibit an organized barrier-like structure at the tumor-stroma boundary, separating tumors from immune/stromal regions. dMMR tumors show disorganized clusters. dMMR SD patients (low anti-PD1 clinical benefit) display organized tumor-stroma boundaries like ICB-insensitive pMMR tumors - Immune cell clusters accumulated near (±500 µm) the tumor-stroma boundary in dMMR but are discontinuous and stromal-localized (≤500 µm) in pMMR. - Treatment-naïve dMMR have elevated *CXCL13* expression in CD4^+^/CD8^+^ T cells vs*.* pMMR. Post-treatment, PR/CR patients show higher *CXCL13* ^+^ CD4^+^ and CD8^+^ T cell proportions than SD patients - These *CXCL13* ^+^ T cells colocalize and interact with *LAMP3* ^+^ DCs via PD-1/PD-L1 at the tumor-stroma boundary in treatment-naïve dMMR and anti-PD1-treated PR/CR.	Feng et al. (2024)
LCRC		
Macrophages	- High expression of pro-inflammatory molecules from macrophages in LCRC is identified - During malignant progression of LCRC and RCRC, there is an increase in exhausted CD8^+^ T cells and *SPP1* ^+^ macrophages	Hu et al. (2025)
	- M1-like macrophages are predominant in LCRC and demonstrate functional enrichment in inflammatory-related pathways, transcriptional regulation, and B-cell receptor signaling pathways	Ji et al. (2024)
T cells	- T cells within LCRC predominantly display a low differentiation and naïve state. Immunofluorescence staining identified that LCRC is enriched for CD4^+^ T cells - Within CD4^+^ T cell populations, exhaustion related molecules are predominantly expressed in the Treg cell subset, which is associated with immune tolerance	Liu D et al. (2024)
	- T cell functional signaling molecules are highly expressed in LCRC. T cells are enriched in stress signaling pathways, suggesting that left sided-specific T cells keep their activation state - CD4^+^ T cell subclusters are enriched in early and middle-stage CRCs with no obvious variation in the distribution between LCRC and RCRC. - CD8^+^ T cells characterize two main functional states: cytotoxicity and exhaustion. Cytotoxic CD8^+^ T cells are significantly enriched in early and middle-stage CRC, whereas exhausted CD8^+^ T cells from late-stage CRC are greater in proportion than cytotoxic T cells – such a shift in functional properties of T cells is detected regardless of tumor sidedness	Hu et al. (2025)
	- Tumor sidedness has a minimal effect on the ICL/ICRs expression pattern and is not an effective predictor for immune checkpoint blockade-based immunotherapies - CD4^+^ T cells in LCRC have significantly higher levels of oxidative phosphorylation and the TCA cycle than those in RCRC, suggesting a higher T cell function in LCRC (similar results were also observed in CD8^+^ T cells) - Tumor sidedness has minimal effect on cell-to-cell communication	X. Liu et al. (2022)
	- The proportion of CD4^+^ T is higher in LCRC - Tregs with high expression of *IL2RA*, *FOXP3*, and *CTLA4* account for the highest proportion of CD4^+^ T cell subsets and are increased in LCRC - CD8^+^ T cells in LCRC are at the terminal ends of cytotoxic and exhausted states	Liu B et al. (2024)
B cells	- Tumor samples contain fewer naive B cells than adjacent normal samples, and naive B cells are absent in LCRC samples	Ji et al. (2024)
RCRC		
Monocytes/macrophages	- Anti-inflammatory genes and *SPP1* are enriched in RCRC - During malignant progression of both left- and right-sided CRC, there is a significant increase in the distribution of CD8^+^ T cells exhibiting exhaustion and *SPP1* ^+^ macrophages	Hu et al. (2025)
	- A higher proportion of monocytes was identified in RCRC vs*.* LCRC. Analysis of biological functions of the top variable genes in monocytes demonstrated activation of processes such as leukocyte, mononuclear and lymphocyte proliferation, regulation of leukocyte proliferation, antigen processing and presentation, and MHC protein complex binding - It was identified that *LUCAT1* locus variations were associated with an increase in CRC risk and that LUCAT1 is predominantly expressed in monocytes and neutrophils and is highly expressed in RCRC - Metabolic function differences are identified between *LUCAT1* ^+^ and *LUCAT1* ^−^ monocytes: heightened activity in glycosaminoglycan degradation, ubiquinone and other terpenoid-quinone biosynthesis, and thiamine metabolism - *LUCAT1* ^+^ monocytes are involved in biological processes such as positive regulation of interleukin-6 production, the NF-kB signaling pathway, pattern recognition receptor activity, cellular response to lipopolysaccharide, and the integrin complex	Shang et al. (2024)
	- M2-like macrophages are more prevalent in RCRC (validated in several datasets). They interact predominantly with leukocytes	Ji et al. (2024)
	- *SPP1* ^+^ macrophages are predominantly located in RCRC (confirmed by IHC analysis). The genes upregulated in these cells are enriched in the positive regulation of hypoxia, angiogenesis, and EMT pathway - Patients with higher infiltration of *SPP1* ^+^ macrophages cells have shorter 5-year OS rates (confirmed in several datasets)	Liu B et al. (2024)
T cells	- T cells within RCRC exhibit a highly differentiated and recently activated state. Immunofluorescence staining for CD4 and CD8 markers identified that RCRC is enriched for CD8^+^ T cells - A subpopulation of T cells in RCRC are in an exhaustion state: the increased presence of these cells frequently signifies a positive reaction of the immune system towards the tumor and may result in a more favorable prognosis when utilized together with immune checkpoint therapy	Liu D et al. (2024)
	- Early-stage CD8^+^ T cells are predominantly distributed in RCRC - *CD161* ^+^ CD8^+^ T cells are enriched in RCRC and are linked to lower OS of patients	Liu B et al. (2024)
B cells	- Tumor samples contain fewer naive B cells than adjacent normal samples. *CD79B, CD20, TNFRSF17*, and *SMIM14* B-cell specific genes are associated with a favorable prognosis in RCRC - *CD20* ^+^ B cells might serve as prognostic indicators for CRC patients (TCGA and IHC confirmed), particularly RCRC, and modulate the TME through interactions with other immune cells - CRC patients with high intratumoral densities of CD20 and CD3 subsets exhibit prolonged survival vs*.* those with low densities of both subsets (particularly significant in patients with RCRC). IHC staining results confirm positive correlation between the densities of tumor-infiltrating B cells and improved survival in patients with CRC, with the strongest association observed in patients with RCRC - CT26 murine models show that anti-PD-1 treatment effectively inhibited tumor progression; the depletion of CD20^+^ B cells reversed this inhibition. Patient-derived organoids demonstrate that the addition of anti-CD20 diminishes the tumor-killing effect of anti-PD-1 treatment, demonstrated by flow cytometry assays	Ji et al. (2024)

The role of tumor-associated macrophages (TAMs) in CRC is defined by functional heterogeneity in a number of studies employing sc-seq. Zhang et al. identified two transcriptionally distinct TAM populations with opposing roles: 1) C-chain polypeptide of serum complement subcomponent C1q-positive (*C1QC* ^+^) TAMs expressing genes related to phagocytosis and antigen presentation and predicted to interact primarily with immune cells (particularly T cells) via enriched immune cell recruitment CXCL10-CXCR3 signaling and 2) osteopontin-positive (*SPP1* ^*+*^) TAMs upregulating regulators of angiogenesis and metastatic pathways, engaging with CAFs and myofibroblasts and participating in ECM degradation SDC2-MMP2 signaling crosstalk with endothelial cells (Zhang et al., 2020). This TAM plasticity represents a clinical challenge, as demonstrated in Renca tumor–bearing mice, where anti-colony stimulating factor 1 receptor (CSF1R) therapy - which disrupts a pathway essential for macrophage development, differentiation, and survival (O’Brien et al., 2021; Wen et al., 2023) - reduced total TAM numbers but revealed a treatment-resistant TAM subset (Zhang et al., 2020). These resistant TAMs preferentially express angiogenic (*VEGFA*) and immunosuppressive (*CD274*, *ARG1*) genes and promote tumor vascularization. Hence, the TME in CRC is composed of specialized TAM subpopulations with opposing roles: immune coordination (*C1QC* ^+^) with anti-tumor functions versus stromal activation (*SPP1* ^+^) involved in tumor progression signaling. Notably, resistance to TAM-depleting therapies arises from a vasculature-promoting TAM subset, suggesting the need for macrophage subtype-specific targeting strategies in CRC treatment and, potentially, adjust therapy based on tumor sidedness. Further complexity of the immune cell niche is revealed by the gut’s highly organized macrophage network, where distinct subsets are specific to their anatomical niche (Domanska et al., 2022). Lamina propria macrophages (LpMs) in the subepithelial region include proinflammatory and antigen-presenting/phagocytic subsets highly expressing proinflammatory (e.g., *S100A9*) and antigen presentation genes (e.g., *HLA-DRA*). Notably, MHC II-high LpM subsets interact with T cells via both immune cell co-stimulatory and co-inhibitory pairs like CD28/CTLA4-CD80/CD86 and recruit CD8^+^ T cells via chemokine CXCR3 ligands. Furthermore, submucosal macrophages (SmMs), particularly lymphatic vessel endothelial hyaluronan receptor 1-positive (*LYVE1* ^*+*^ *)* SmMs, exhibit low antigen presentation but high chemotactic and tissue-protective capacity. Finally, muscularis macrophages (MMs) express homeostatic genes and engage in the crosstalk with neurons: neuron-derived Notch ligands DLL1/DLL3/JAG2-NOTCH2 and IL34-CSF1R regulate MM survival and differentiation. Overall, such cellular organization suggests that the development of targeted therapeutic approaches accounting for not only immune cell subtypes, but additionally location-specific functions is preferred.

A promising alternative to broad macrophage depletion is the precision targeting of specific immunosuppressive receptors on TAMs. In support of this approach, Huang et al. reported that TAMs upregulate immunosuppressive receptors bearing immunoreceptor tyrosine-based activation motifs (ITAMs) (Huang et al., 2024). These include signal regulatory protein α *(SIRPA)*, which showed significantly higher expression in tumor tissues versus paracancerous/normal tissues. SIRPA/Sirpα elevated levels are associated with tumor progression, as genetically engineered SIRPA^−/−^ mice were demonstrated to have developed smaller subcutaneous MC38 tumors and exhibited prolonged survival, while rechallenged tumors were completely rejected. Mechanistically, Sirpα knockout (KO) reprograms macrophages to recruit T cells to tumors via spleen tyrosine kinase/Bruton tyrosine kinase-dependent (SYK/BTK-dependent) C-C motif chemokine ligand 8 (CCL8) secretion, thereby activating immune response (Huang et al., 2024). These findings demonstrate that precision targeting of a subset of TAM receptors/signaling pathways can effectively remodel the TME, suggesting a potential therapeutic targeting strategy.

The T cell landscape in CRC is equally as complex, with specific subsets correlating with differing patient outcomes. On one hand, cytotoxic CD4^+^ T cells are characterized as cells expressing high levels of effector molecules and co-inhibitory molecules, such as programmed cell death protein 1 (PD-1), pointing to their tumor-suppressing functions (Masuda et al., 2022). Additionally, among CD8^+^ T cells, a Tem phenotype is associated with a less dysfunctional transcriptomic state and positive clinical outcomes; similar results are identified within Tregs population, where transcription factor Helios-positive (*Helios* ^*+*^) Tregs are associated with improved patient outcomes and present in early-stage cancers. On the other hand, granulysin- and integrin αE-positive *(GNLY* ^*+*^ *CD103* ^*+*^) T cells, found specifically within tumors (and absent in normal colon tissue), demonstrate a loss of cytokine production and show no correlation with positive outcomes in CRC. Furthermore, peripheral Tregs are associated with poor outcomes (independently of stage), express high levels of cyclic ADP ribose hydrolase (CD38) and lymphocyte-activation gene 3 (LAG3), and produce elevated IL-10, suggesting immunosuppressive activity.

Beyond the adaptive immune response, innate immune cells like mast cells (MCs) also play a significant role in CRC signaling. Sc-seq studies have identified that the activated-to-resting MC ratio is significantly altered in CRC, a pattern validated across multiple datasets (Xie et al., 2023). Functional-wise, gene set variation analysis (GSVA) analysis links activated MCs to immunostimulatory TNFα/NFκB signaling and MHC I/II enrichment, while resting MCs are associated with angiogenesis, a process important for tumor growth. Moreover, a high MC gene signature correlates with better survival in TCGA patients, underscoring the biological significance of these findings. When looking at cellular communication, activated MCs exhibit more intercellular interactions (including myeloid, endothelial, epithelial, fibroblast) than resting MCs, and ligand expression for the tumor progression inhibitor KITLG-KIT axis on interacting cells is identified higher for activated MCs. In support of these findings, *in vitro* experiments show that KITLG-activated mast cells demonstrate reduced proliferation and migration, suggesting that KITLG/KIT signaling may inhibit CRC progression and confirming its computational significance.

Importantly, the functional impact of immune cells in CRC is critically dependent not only on the differing subtypes and signaling employed, but additionally on the tumor’s spatial organization. Such cellular organization is shown to be influenced by the mismatch repair (MMR) status (Feng et al., 2024). Studies have revealed that proficient MMR (pMMR) tumors, which are typically poor immunotherapy responders, form an organized barrier separating tumor cells from stromal and immune regions. Deficient MMR (dMMR) tumors, on the other hand, show disorganized clusters. Further research uncovered proximity-dependent (<250 µm) chemokine ligand 13-positive (*CXCL13* ^+^) T cell–lysosomal-associated membrane protein 3-positive *(LAMP3* ^+^) dendritic cell (DC) interactions via PD-1/PD-L1, a signaling axis utilized by DC to attenuate T cell activation (Peng et al., 2020), occurring at the tumor-stroma boundaries in dMMR and anti-PD1-responding (partial response (PR)/complete response (CR)) tumors, highlighting distance-critical signaling (Table 2). These findings highlight the fundamental importance of not only intercellular communications, but additionally spatial interconnection between tumor cells and immune/stromal cells and the distance at which they are able to effectively signal.

Thus, the immune cell subsets display functional duality, which suggests that therapies tailored to tumor spatial location is an option. Spatial architecture fundamentally determines therapy response, and effective targeting requires precision against specific dysfunctional states and communication pathways within the tumor. In conclusion, the CRC immune landscape can be characterized as a dynamic ecosystem defined by functional duality as well as spatial context. The success of targeted approaches for CRC treatment (like anti-SIRPα) demonstrate that future treatment strategies should be precisely designed. Effective targeting may potentially include targeting of specific dysfunctional cell states (such as *SPP1* ^+^ TAMs, peripheral Tregs), disruption of pro-tumor communication pathways (such as SPP1-CD44, CD74-MIF), and should account for the spatial context of the TME, including MMR status which shapes these cellular interactions. Moreover, a targeting approach based on tumor sidedness could significantly enhance their efficacy.

#### 3.1.3 Mechanisms of stromal cell signaling in CRC progression

While immune checkpoint blockade has become a standard of care for many cancers alongside chemotherapy, the stromal niche of the TME remains a mediator promoting tumor growth, metastasis, and CRC therapy resistance (Zhao et al., 2023). Single-cell and spatial transcriptomics studies are now uncovering how specific stromal cell populations are involved in the aforementioned processes, revealing new biomarkers and therapeutic vulnerabilities (summarized in Table 5).

**TABLE 5:** Transcriptomic features of stromal cells in overall and sided CRC progression revealed by single-cell sequencing and spatial transcriptomics.

Cell type	Key findings	Source
Overall CRC		
Fibroblasts	- Several CAF subsets with opposing clinical outcomes are identified: *PI16* ^*+*^ */SLIT2* ^+^/*ADAM28* ^+^ subset enriched in responders and a tumor-promoting subset *BMP4* ^*+*^ */FAP* ^*+*^ */MMP1* ^*+*^ enriched in non-responders - *FAP* ^*+*^ CAFs enhanced tumor EMT through MIR4435-2HG associating with worse neoadjuvant chemotherapy outcomes - *PI16* ^*+*^ */SLIT2* ^+^ CAFs are identified in the responsive patient group and demonstrate immune-promoting properties such as immune cell recruitment and activation	Qin et al. (2023)
	- *CTHRC1* ^*+*^ fibroblasts are found only in tumors (prevalence increasing from early to advanced cancer stages). This group is defined by upregulated ECM remodeling genes (*POSTN, MMP11, TGF-β1*) and is more active in promoting EMT and angiogenesis than other fibroblasts - Spatial analysis demonstrates that *CTHRC1* ^*+*^ fibroblasts co-locate with malignant epithelial cells in a niche featuring active EMT, high immunosuppressive immune cell infiltration (*TREM2* ^*+*^ */SPP1* ^*+*^ macrophages, Tregs, Th17 cells), and exclusion of CD8^+^ T cells - *In vitro*, *CTHRC1* ^*+*^ CAFs secrete WNT5A, which upregulates the *MSLN* gene in CRC cells. This effect is abolished by WNT5A inhibition or *CTHRC1* knockdown - *CTHRC1* ^*+*^ CAFs worsen prognosis by creating an immunosuppressive, pro-invasive niche and directly upregulating *MSLN* in tumor cells via WNT5A secretion	Lu et al. (2025)
	- A positive correlation is found between stromal and myeloid cell infiltration, and high levels of both are associated with worse overall and progression-free survival - *FAP* ^*+*^ fibroblasts and *SPP1* ^*+*^ macrophages are enriched in tumors and their high infiltration correlates with shorter survival, advanced cancer stage, and MSI-H status - Spatial transcriptomics revealed co-localization of *FAP* ^*+*^ fibroblasts and *SPP1* ^*+*^ macrophages around malignant cells, with activated pathways for ECM organization and TGF-β response - Tumors with high *FAP* ^*+*^ */SPP1* ^*+*^ infiltration show enriched signatures for hypoxia, EMT, TNFα signaling, and IL2/STAT5 signaling and such infiltration correlates with resistance to immunotherapy	Qi et al. (2022)
Endothelial cells	- Tumor ECs undergo gene expression changes, switching to a pro-angiogenic profile (upregulated VEGFA-VEGFR2, ECM genes like *COL4A1*, *SPARC*) while downregulating antigen presentation pathways (MHC II) - Among identified EC subsets, tip cells exhibit the highest expression of growth factors (*PGF, ANGPT2*) and receptors (*KDR/VEGFR2, FLT1/VEGFR1*). Tip cells are the primary EC subset responsible for the loss of MHC I and MHC II expression - A VEGFA-VEGFR-ESM1 positive feedback loop maintains tip cell identity. *In vitro*, tip cell-secreted ESM1 protein directly upregulates VEGFA expression in CRC cells, perpetuating the cycle. Spatial transcriptomics confirms tip cells co-localize with stromal VEGF/VEGFR signaling hubs - Tip cell abundance is linked to CRC tumorigenesis, progression, and poor prognosis. Successful anti-PD-1 immunotherapy significantly reduces tip cell populations, highlighting their role in treatment resistance	Xie et al. (2024)
LCRC		
Fibroblasts	Sc-seq revealed greater tumor heterogeneity in LCRC vs. RCRC, with the difference driven primarily by non-immune cells. Fibroblasts in LCRC highly express *RHOB*, a gene controlling cell growth and migration - Functional enrichment analysis, especially in Stage III tumors, showed LCRC fibroblasts are uniquely pro-tumorigenic. They are characterized by strong activation of TGF-β response, promoting stroma deposition and CAF formation and activation of tumor progression pathways like cell proliferation and angiogenesis - *MYH11* ^+^ CAFs are highly enriched in LCRC and exhibit aggressive features: 1) express pro-tumor genes *RAMP1* (induces T-cell exhaustion, promotes angiogenesis), *MYLK*, and *MYH11* (drive tumor cell contraction and migration), 2) have elevated scores for proliferation, stemness, and hypoxia and have 3) activated muscle-related pathways, suggesting a role in facilitating tumor cell migration and invasion - These *MYH11* ^+^ CAFs predominantly interact with myeloid cells (macrophages), shaping an immunosuppressive microenvironment	C. Wang et al. (2024)
RCRC		
Fibroblasts	- CAFs in RCRC uniquely express AGR2, a protein that potentiates VEGF and FGF2 signaling to drive endothelial cell proliferation and angiogenesis - Right-sided CAFs are characterized by the overexpression of *REG4*, which exacerbates tumor invasion, and transcription factors *E2F1* and *FOXF1*, which promote rapid tumor growth by activating the “cell cycle” and “apoptosis” pathways, with *FOXF1* also contributing to neovascularization	Hu et al. (2025)

The CAF population is not uniform, but rather consists of functionally divergent subsets that influence patient response to administered therapy. One such example involves the study of post-neoadjuvant chemotherapy (NAC) biopsies (Qin et al., 2023). Two clinically opposing CAF subsets a favorable subset (*PI16* ^*+*^ */SLIT2* ^*+*^ */ADAM28* ^+^), enriched in responders (CR/PR), regulating cell differentiation and immunoregulation as well as a tumor-promoting subset (*BMP4* ^*+*^ */FAP* ^*+*^ */MMP1* ^+^), enriched in non-responders (NR), upregulating collagens, matrix metalloproteinases (MMPs), and WNT signaling to drive ECM remodeling and progression. Functionally, fibroblast activation protein α-positive (FAP^*+*^) CAFs promote epithelial-mesenchymal transition (EMT) and therapy resistance via long non-coding RNA MIR4435-2HG, a molecule previously associated with cancer progression including CRC (Ghasemian et al., 2022), while peptidase inhibitor 16-/slit guidance ligand 2-positive (*PI16* ^*+*^ */SLIT2* ^*+*^) progenitor CAFs foster immunity and inhibit tumor progression by recruiting and activating T cells. This occurs through mechanisms like stromal derived factor 1 (CXCL12)-mediated recruitment of CD8^+^ Tem over Tregs and decorin (DCN)-enhanced antigen presentation by DCs for spatial T cell immunostimulatory activation. Thus, these findings establish specific CAF populations as biomarkers of clinical outcome and identify novel mechanisms and targets for modulating the TME to overcome therapy resistance.

In support of the aforementioned findings, a particularly aggressive pro-tumor CAF population - the collagen triple helix repeat containing 1-positive (*CTHRC1* ^+^) fibroblasts - was identified: a tumor-exclusive subset whose abundance increases with cancer progression and correlates with poor patient prognosis (Lu et al., 2025). Functionally, this group is characterized by a potent ECM-remodeling signature (e.g., expression of *POSTN, MMP11, TGF-β1*) and exhibits heightened transcriptional activity in promoting EMT and angiogenesis. In an attempt to arrange the identified cell population/signaling characteristics spatially within the tumor, the authors employed spatial transcriptomics analysis, revealing that *CTHRC1* ^+^ CAFs co-localize with malignant epithelial cells to form a specialized niche characterized by active EMT, high immunosuppression (featuring *TREM2* ^*+*^ */SPP1* ^*+*^ macrophages, Tregs, and Th17 cells), and exclusion of CD8^+^ T cells. Within this niche, they exhibit the strongest interactions with tumor cells, primarily through outgoing signals via WNT, Notch, and ECM-adhesion pathways (for example, COL1A1-ITGB1, LAMA1-ITGA3). Functionally, these pathways are known to enhance tumor cell migration and invasion through WNT5A-LRP6/FZD3 and THBS1-ITGAV ligand-receptor signaling pathways. Crucially, *in vitro* validation confirms that *CTHRC1* ^+^ CAFs secrete WNT5A, which upregulates the mesothelin (*MSLN*) gene in CRC tumor cells - an effect abolished by WNT5A inhibition or *CTHRC1* knockdown. Collectively, these findings establish that *CTHRC1* ^*+*^ CAFs drive poor outcomes by creating an immunosuppressive, pro-invasive niche and directly promoting tumor aggressiveness via a CTHRC1-WNT5A-MSLN signaling axis.

The negative impact of pro-tumor CAFs is further supported by sc-seq studies revealing the extensive collaboration of CAFs with other cells within the TME. A potent stromal-myeloid network between *FAP* ^*+*^ fibroblasts and *SPP1* ^*+*^ macrophages is detected, with both populations linked to advanced stage, MSI-H status, and reduced patient survival (Qi et al., 2022). These cells co-localize in hypoxic tumor regions and are predicted to engage in direct adhesive interactions (e.g., COL1A1-ITGB1) and paracrine signaling via TGF-β, WNT5A-FZD2, and CCL3-CCR5 axes, promoting recruitment and pro-inflammatory activity of *SPP1* ^*+*^ macrophages. Ultimately, this synergistic *FAP* ^*+*^ */SPP1* ^*+*^ interaction promotes an immunosuppressive, pro-tumorigenic niche that is linked to immunotherapy resistance. Thus, these findings highlight the stromal-myeloid crosstalk as a determinant of patient outcomes and a potential therapeutic target.

Such a collaborative cellular interaction network extends beyond CAFs to the tumor vasculature. Tumor endothelial cells (ECs) in CRC undergo transcriptional reprogramming toward a pro-angiogenic phenotype, upregulating VEGFA-VEGFR2 signaling and ECM genes (e.g., *COL4A1, SPARC*) while downregulating antigen presentation molecules (MHC I/II) (Xie et al., 2024). Among EC subsets, tip cells dominate this pro-angiogenic response, showing high expression of growth factors (*PGF, ANGPT2*) and receptors (*VEGFR, FLT1*). Notably, these tip cells not only dominate the angiogenic response, but are also the primary mediators of MHC loss on the tumor vasculature, thereby actively facilitating immune evasion. Mechanistically, their activity is sustained by a VEGFA–VEGFR2–ESM1 positive feedback loop (based on *in vitro* data): VEGFA - secreted by myeloid and epithelial cells - activates tip cell VEGFR2, promoting ESM1 release that subsequently upregulates VEGFA in tumor cells, sustaining angiogenesis and promoting tumor growth. These findings are further supported by spatial transcriptomics data, demonstrating the presence of tip cells within stromal VEGF signaling niches. Moreover, the clinical relevance of tip cells is underscored by their correlation with poor prognosis and their reduction following successful anti-PD-1 therapy. Hence, these findings highlight them as a promising therapeutic target.

In conclusion, sc-seq and spatial transcriptomic studies reveal that the CRC TME is regulated by functionally diverse stromal cells that critically influence the clinical outcomes of patients. Novel sc-seq findings demonstrate the functional differences in CAFs, with immunogenic subsets (e.g., *PI16* ^*+*^ */SLIT2* ^+^) promoting anti-tumor immunity and positive therapy response, while pro-tumor subsets (e.g., *FAP* ^*+*^ */CTHRC1* ^+^) drive ECM remodeling, immunosuppression, and invasion through pathways like WNT and TGF-β. Importantly, pro-tumor CAFs form spatial niches with immunosuppressive *SPP1* ^*+*^ macrophages and pro-angiogenic endothelial tip cells, collectively promoting an immune-excluded, pro-metastatic TME. The signaling within this stromal network - mediated by ligand-receptor pairs promoting cell migration and invasion such as WNT5A-LRP6 and COL1A1-ITGB1 - correlates with disease progression, immune evasion, and resistance to chemo- and immunotherapy. Thus, targeting these stromal interactions represents a promising strategy for overcoming treatment resistance and tumor progression in CRC.

### 3.2 Single-cell sequencing for analysis of total CRC metastasis mechanisms

Despite extensive research in the field of CRC, distant metastasis remains the leading cause of death and a substantial clinical challenge. Similar to primary CRC tumors, metastases are composed of and signaled by not only cancer cells, but additionally the TME. A key driver of metastasis is the formation of a “pre-metastatic niche” (PMN) (Li et al., 2025). This PMN is characterized by several features, including immunosuppression, inflammation, angiogenesis/vascular permeability, lymphangiogenesis, organogenesis, and metabolic reprogramming. Together, these factors enable the seeding and survival of disseminated tumor cells. Therefore, signaling from the TME is as crucial as signaling from cancer cells in promoting CRC metastasis. Given the importance of this process, we have dedicated a section of this review to compiling how single-cell technologies are helping to elucidate its underlying mechanisms for individual cell types and signaling hubs in tumor cells and the TME.

#### 3.2.1 Mechanisms of tumor cell signaling in CRC metastasis

Upon colonizing the liver, CRC cells transcriptionally reprogram themselves to evade immune detection: liver metastases are characterized by a downregulation of genes responsible for immune-related processes, including neutrophil activation, response to TNF, myeloid leukocyte migration, and granulocyte chemotaxis, suggesting a shift toward reduced immunogenicity (Che et al., 2021). These metastases properties are accompanied by a decrease in the rich cellular crosstalk seen in primary tumors. Rather than utilizing extensive cell-cell communication, metastatic tumor cells are predicted to employ a dominant immune-evading signaling axis through the upregulation of the “do not eat me” ligand CD47 (Wu et al., 2022). This interaction is further confirmed by immunohistochemical (IHC) analysis, which revealed that CD47^*+*^ tumor cells locate in proximity to SIRPA^*+*^ macrophages.

Thus, CRC liver metastasis involves a shift in cancer cell behavior toward immune silence and reduced intercellular interactions, necessitating specialized treatment strategies. Furthermore, the CD47-SIRPA axis is identified as a promising therapeutic target for metastatic CRC.

#### 3.2.2 Mechanisms of immune cell signaling in CRC metastasis

Single-cell analyses reveal that liver metastases are structurally distinct, supporting a niche where altered macrophage and T cell populations regulate immune evasion and therapy resistance processes. One of the features of the metastatic niche is the dominance of immunosuppressive macrophage populations. In support of this, liver metastases were shown to be enriched with *SPP1* ^*+*^ and *MRC1* ^*+*^ *CCL18* ^*+*^ macrophages (IHC confirmed), which exhibit a M2-polarized phenotype with high expression of anti-inflammatory and pro-M2 conversion genes (*APOE* and *MARCO*) and a metabolic shift toward amino acid metabolism, a process tightly linked to tumor outgrowth, metastasis and therapeutic resistance through tight regulation of immune cell activity (Wu et al., 2022; Yang et al., 2023). These macrophages are reprogrammed by metastatic tumor cells themselves, as detected by cell-cell communication analysis, thus revealing the potential mechanism for the formation of a supportive PMN.

Specialization of TAMs in metastases is further evidenced by the redistribution of macrophage subsets. On one hand, primary CRC tumors harbor MHC-low TAMs with impaired antigen presentation, liver metastases are enriched with *THBS1* ^+^ MHC-low TAMs that express pro-angiogenic factors and genes like *THBS1* and *MARCO*, supporting tumor progression (Che et al., 2021). A constant across both sites is the presence of lipid-associated macrophages (LAMs), characterized by increased lipid metabolism, ECM degradation, and complement activation. Their persistence across both primary and metastatic sites suggests a novel location-independent mechanism for the promotion of tumor survival. However, on the other hand, TAMs in the liver communicate less frequently with other cell subtypes when compared to primary tumors, especially with CAFs (Che et al., 2021). This divergence may reflect the distinct ecological pressures at each site. For instance, primary tumors require a dense, supportive stroma to grow and initiate invasion, a process dependent on close CAF interactions. In contrast, metastatic cells, having already completed the invasion and dissemination process, may evolve toward greater self-reliance or shift their dependency to local liver-specific cell types, with their survival “strategy” primarily being immune evasion rather than stromal construction.

Importantly, the administration of chemotherapy remodels this myeloid landscape, although its impact is complex. In one study, it was identified that NAC reverses the immunosuppressive state in responsive patients by reducing the amount of suppressive macrophages and slowing their metabolism (Wu et al., 2022). In contrast, another study identified that chemotherapy reprograms TAMs away from activated states seen in untreated tumors (including pro-inflammatory *IL1B* ^*+*^ and immunosuppressive *CXCL10* ^*+*^ subsets) toward less active, immature, and uniformly immunosuppressive phenotypes (Che et al., 2021).

When looking at T cells populations, they are equally dynamic and responsive to both primary/metastatic location and therapy. Treatment-naïve tumors, according to sc-seq findings, contain diverse CD8^+^ T populations, including effector and exhausted T cells, whereas metastatic sites accumulate primarily dysfunctional or exhausted CD8^+^ T cells (Che et al., 2021). A critical finding is that chemotherapy inhibits the accumulation of dysfunctional CD8^+^ T cells in both primary and metastatic sites, as confirmed by flow cytometry and immunofluorescence analysis, while also reducing Treg abundance in primary tumors (although Treg levels remained comparable in liver metastases). Furthermore, differentiation trajectory analysis confirms that dysfunctional CD8^+^ T cells predominated in untreated tumors, while Tregs were persistent regardless of treatment. Mechanistically, proliferating (*MKI67* ^+^) CD8^+^ T cells and exhausted T cells exhibit rich ligand-receptor interaction profiles, communicating with stromal (CAFs, endothelial cells) and immune cells. These findings suggest that for successful chemotherapy of metastatic CRC, the dysfunctional immune cell niche should be reactivated.

Overall, these findings depict a liver metastatic TME that is spatially organized, immunosuppressive and formed by metastatic cells to ensure their survival. This state is maintained by specifically reprogrammed macrophages (*SPP1* ^+^, *MRC1* ^*+*^ *CCL18* ^*+*^ *, THBS1* ^+^) and additionally LAM populations. The mentioned findings carry significant clinical implications. Firstly, the identified macrophage subpopulations represent as novel therapeutic targets for CRC metastases. Secondly, the composition of the TME, in particular macrophage subtypes and T cell dysfunction, could be looked at as biomarkers for predicting chemotherapy response. Lastly, the immunological difference between primary and metastatic sites suggest the need for location-optimized treatment. In conclusion, the liver metastatic TME in CRC is distinct from the primary tumor site and characterized by a more immunosuppressive state that is regulated by reprogrammed macrophage populations. This TME supports metastasis progression, although chemotherapy can reverse this state in responsive patients.

#### 3.2.3 Mechanisms of stromal cell signaling in CRC metastasis

The identification of CAFs is now shifting from a uniform population to distinct subsets whose prevalence is altered by therapeutic intervention. In support of this, a study identified three major groups of CAFs in CRC tumors - secretory, ECM-remodeling and contractile (Che et al., 2021). Notably, out of the mentioned CAF groups, treatment-naïve tumors include ECM-remodeling CAFs that support ECM organization and the activity of collagen metabolism, supporting tumor ECM, whereas chemotherapy-treated primary and metastatic tumors exhibit a shift towards contractile CAF niche formation, enriched for pathways regulating muscle cell differentiation, T cell activation and tumor proliferation. These activated myofibroblasts further reinforce a treatment-resistant state by activating JAG1/NOTCH pathway in an autocrine and a paracrine manner with endothelial cells, promoting blood vessel development and differentiation. These findings indicate that chemotherapy can drive a CAF switch to a pro-proliferative and therapy-resistant state both in primary and metastatic tumors, highlighting the need for stromal-targeting adjuvants.

Apart from resident fibroblasts in CRC metastases, pathways involving trans-differentiation of tumor cells into CAFs are noted. Comparative analysis of primary tumors and metastases has revealed that the latter are enriched in processes regulating EMT, angiogenesis and TGF-β signaling (Yang et al., 2024). Notably, a specific subtype of tumor cells enriched in tumor metastases are able to undergo EMT and further differentiate into growth-regulated oncogene-α*-positive (CXCL1* ^+^) CAFs, which subsequently mature into secreted frizzled-related protein 2*-*positive (*SFRP2* ^+^) CAFs. The master regulator of this phenotypic switch is the transcriptional factor basic helix-loop-helix protein 40 (BHLHE40), whose expression is associated with poor patient prognosis. Functional studies confirmed that BHLHE40 drives tumor cell proliferation, migration, invasion and liver metastasis both *in vitro* and *in vivo.* This discovery of a tumor cell-to-CAF axis demonstrates a novel mechanism of metastatic adaptation and positions BHLHE40 and its downstream pathways as a target for therapeutic intervention to prevent metastatic seeding.

As mentioned previously, the functions of the TME depend not only on its cellular components, but additionally on the specific intercellular communication networks. A key pro-metastatic axis identified with the help of single-cell technologies involves a chemoresistant, chemokine receptor type 4-positive *(CXCR4* ^*+*^ *)* and glutathione peroxidase 4-positive *(GPX4* ^+^) tumor cell subpopulation with notable enhanced migratory capacity and ferroptosis resistance, as well as a *NOX4* ^*+*^ *TGFB1* ^*+*^ *CXCL12* ^+^ CAF population involved in immune regulatory pathways (Z. Wu et al., 2025). The survival of this niche depends on the chemokine-dependent CXCL12-CXCR4 ligand-receptor signaling axis, where CAF-derived CXCL12 binds to CXCR4 on tumor cells, fostering a tumor-promoting niche that suppresses ferroptosis and promotes survival. Targeting this interdependence, a dual-targeting TME-modulating nanoplatform CCM-FSS&CHM-ABI was developed. This strategy simultaneously induces ferroptosis in tumor cells and reprograms supportive CAFs (composed of ferroptosis inducer sulfasalazine and SN38 prodrugs, and CHM-ABI, a chelation product of azithromycin and baohuoside I). This coordinated approach successfully drives CAFs into a quiescent state, while concurrently inducing ferroptotic cell death in a co-culture model system with tumor cells. This coordinated targeting significantly suppresses primary tumor growth and liver metastases *in vivo*, demonstrating the potent efficacy of simultaneously targeting both the tumor and its stromal niche.

Overall, CRC metastasis is driven by a dynamic TME. Importantly, CAFs are active participants in promoting metastasis and therapy resistance. The stromal TME composition can be influenced by chemotherapy, resulting in a more aggressive and therapy-resistant tumor state due to contractile CAFs promotion of muscle cell differentiation, T cell activation and tumor cell proliferation. Moreover, tumor cells can differentiate into *SFRP2* ^+^ CAFs, thereby promoting tumor growth and metastasis. Cell-cell communication is also of importance in shaping the TME, where metastatic survival depends on specific signaling (through CXCL12-CXCR4) between *GPX4* ^+^ tumor and *NOX4* ^*+*^ *TGFB1* ^*+*^ *CXCL12* ^+^ CAFs. Importantly, dual therapeutic targeting of the aforementioned cell-cell interaction successfully suppressed liver metastases. Thus, for metastasis prevention, successful treatment requires targeting not only tumor cells, but additionally the reprogramming of the TME, as well as their interactions.

## 4 Insights from single-cell sequencing in left- and right-sided colorectal cancer

**FIGURE 1:**
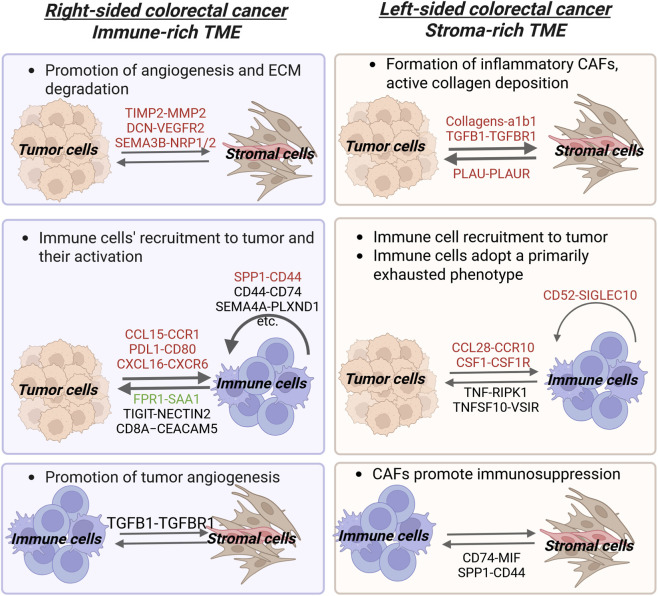
Ligand-Receptor interactions in Left- and Right-sided Colorectal Cancer Microenvironment. This figure depicts intercellular crosstalk between tumor, immune, and stromal cells. Ligand-receptor pairs are shown above the arrows, indicating the direction of signaling between cell populations. The pairs are color-coded by their function: red for tumor-promoting, green for tumor-suppressive, and black for pairs with no indicated function (for more details refer to Table 2 and the text). Generally, LCRC is characterized by a more reactive tumor stroma and a growth factor-driven TME, whereas RCRC has a predominantly immune cell-signaling TME. In left-sided colorectal cancer, bidirectional tumor cells-CAFs signaling promotes the formation of inflammatory CAFs and tumor growth respectively. Concurrently, crosstalk between B cells myeloid cells inhibits immune activation. Specific immune-tumor interactions (such as those between tumor cells and B cells or *PLTP* ^*+*^ macrophages) further contribute to an immunosuppressive microenvironment. In right-sided tumors, crosstalk between tumor cells and fibroblasts/endothelial cells promotes angiogenesis and ECM remodeling. Furthermore, bidirectional signaling between IS^high^ tumor cells and *SPP1* ^+^ macrophages/*CD161* ^+^CD8^+^ innate-like T cells impairs immune function by reducing the efficiency of antigen presentation and T cell activation, while also altering cytokine production correspondently. Additional immune cell crosstalk, such as between *SPP1* ^+^ macrophages and *CD161* ^*+*^CD8^+^ innate like T cells, further dampens cytotoxic T cell capability (Created with Biorender.com).

While studies have given insights into the molecular mechanisms of overall CRC heterogeneity, the distinct cellular ecosystems underlying its most significant clinical classification - left- versus right-sided origin - remain incompletely defined. To elucidate these differences, we have compiled the current sc-seq data on the unique pathogenic mechanisms characterizing left-and right-sided CRC tumors, making an emphasis on cell-cell interactions (Table 2; Figure 1).

### 4.1 Single-cell sequencing to elucidate tumor progression in left- and right-sided colorectal cancer

#### 4.1.1 Mechanisms of tumor cell signaling in left- and right-sided colorectal cancer progression

**FIGURE 2:**
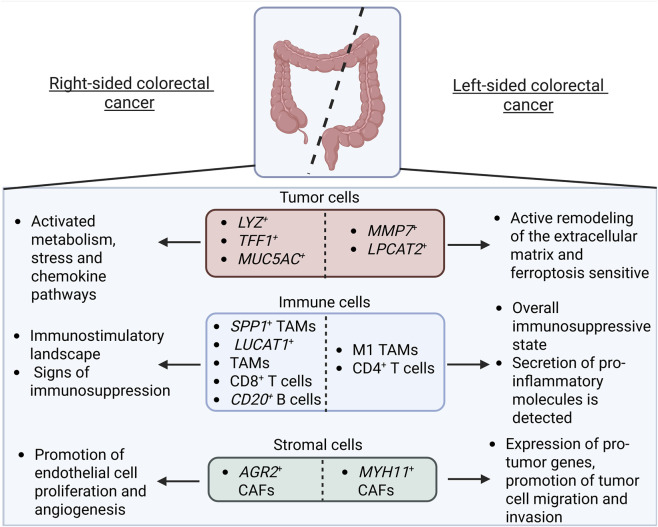
Comparative Overview of the Tumor Cell Subtypes and the Tumor Microenvironment in Right-Sided vs. Left-Sided Colorectal Cancer. Left-sided colorectal cancer is characterized by *MMP7* ^+^ and *LPCAT2* ^+^ tumor cells that remodel the extracellular matrix and are sensitive to ferroptosis. The immune landscape, namely CD4^+^ T cells, forms an overall immunosuppressive state (although some studies point to the secretion of pro-inflammatory molecules by macrophage M1-like subsets). The stromal compartment is characterized by CAFs that promote tumor cell migration and invasion. Right-sided colorectal cancer *LYZ* ^+^, *TFF1* ^+^, and *MUC5AC* ^+^ tumor cells, in turn, are metabolically active and upregulate stress and chemokine-related pathways. Immune cells, namely CD8^+^ T cells and *CD20* ^+^ B cells, are immunostimulatory (though subsets of *SPP1* ^+^ and *LUCAT1* ^+^ TAMs exhibit immunosuppressive properties). CAFs, in turn, promote endothelial cell proliferation and angiogenesis (Created with Biorender.com).

Sc-seq technology has identified that LCRC and RCRC are driven by different core molecular programs (Liu B et al., 2024) (Table 3; Figure 2). The malignant cells in LCRC are frequently defined by a proliferation stemness (PS) program. The latter activates genes linked to proliferation and stem-like properties (for example, *UBE2C*, *MYBL2* and *ALDH1A1*), and these proliferative tumor cells are often found in highly glycolytic niches within the tumor and are supported by specific immune cells, including *PLTP* ^+^ macrophages, activated Tregs, and *LAYN* ^*+*^ CD8^+^ cells. In turn, RCRC is characterized by an immune-secretory (IS) program. Here, the tumor cells elevate expression of immune signaling MHC II molecules (*CD74*, *HLA-DRB1*, *HLA-DPA1*) and immune-regulating secretory proteins (*REG4*, *AGR2*, *AGR3*), with *AGR2* and *REG4* specifically upregulated in RCRC. These tumor cells reside near hypoxic tumor cores and are surrounded by a different set of immune cells such as *SPP1* ^+^ macrophages and *CD161* ^+^ CD8 T cells. Hence, these findings demonstrate that LCRC and RCRC are molecularly distinct diseases driven by differing molecular programs (PS and IS), each associated with unique metabolic states, specific secreted factors/gene expression, and spatially organized, distinct immune microenvironments. This difference has significant implications for understanding tumor biology and developing sidedness-specific therapies.

The tumorigenic signaling pathways employed by tumor cells also differ by tumor sidedness. For instance, in LCRC, researchers have identified a subgroup of cancer cells enriched for genes involved in maintaining of the gut lining (e.g., *TFF1*, *TFF2*, *AGR3*, *MUC5AC*) (Guo et al., 2022). These cells exhibit enhanced activity in estrogen and ERBB signaling pathways, which helps to explain why LCRC generally responds better to therapies targeting these pathways. Furthermore, LCRC frequently signals through fibroblast-secreted collagen that binds tumor cell α1β1 integrins, as well as through macrophage-secreted TNF that interacts with tumor cell TNF receptors. These findings provide clues to the mechanistic basis for the established greater sensitivity of LCRC to monoclonal antibodies targeting ERBB receptors.

LCRC may additionally harbor tumor cells with features that may, by contrast to previously mentioned findings, inhibit tumor progression. In a study of Cao et al., LCRC lower malignancy score is linked to lysophosphatidylcholine acyltransferase 2-positive (*LPCAT2* ^+^) tumor cells (Cao et al., 2024), with *LPCAT2* ^+^ tumor cells significantly expanding in the inflammatory phase of a colitis-associated colon cancer mouse model. Functionally, *LPCAT2* overexpression significantly inhibits cell proliferation and colony formation *in vitro*, while repressing tumor growth and Ki67 expression *in vivo*. Conversely, *LPCAT2* downregulation enhances proliferative capacity *in vitro* and stimulates tumor growth and Ki67 *in vivo*. Furthermore, findings uncovered that *LPCAT2* acts as a tumor suppressor through a novel ferroptosis-inducing PRMT1/SLC7A11 signaling axis. This pathway represents a promising therapeutic vulnerability unique to left-sided tumors.

In contrast, RCRC is characterized by tumor cells enriched in metabolic and inflammatory hallmarks, with glycolysis being particularly prominent in mucinous adenocarcinoma (MAC) (Xu et al., 2025). This enrichment points to a strong dependence on glycolytic metabolism in RCRC tumor cells, especially in the MAC subtype, concurrent with the findings of Liu B et al. (2024). The metabolic shift towards aerobic glycolysis (the Warburg effect) in MAC renders it potentially vulnerable to glycolytic inhibitors or other metabolic-targeted therapies. Moreover, the enrichment of inflammatory hallmarks in RCRC cancer cells suggests anti-inflammatory agents could modulate the TME.

Further underscoring their molecular differences, LCRC and RCRC tumor cells exhibit distinct functional specializations, including enhanced protein folding in left-sided tumors and increased structural homeostasis in right-sided tumors (Hu et al., 2025). A dominant feature of RCRC is a heightened hypoxic state, driven by elevated HIF1A expression in specific subclusters. This hypoxic ecosystem fosters the emergence of functionally distinct tumor populations. For example, hypoxia-responsive RCRC subclusters include *LYZ* ^+^ cells that activate metabolic pathways, *TFF1* ^+^ cells engaged in stress response and *MUC5AC* ^+^ cells upregulating chemokines. In contrast, advanced LCRC is characterized by *MMP7* ^+^ cancer cells expressing ECM remodeling genes and possess significantly higher metastatic potential. Other subpopulations, like *ATP5MC2* ^+^ cells enriched in ATP synthesis, are found across early- and middle-stage CRCs regardless of sidedness. These specialized tumor subpopulations actively shape the TME through specific intercellular ligand-receptor interactions. These include immunosuppressive, angiogenic and migratory interactions through specific tumor cell-TME ligand-receptor pairs (for instance, CCL28-CCR10 with B cells, PDGFA-PDGFRA/PDGFRB with CAFs, and PLAU-PLAUR with TAMs). These findings highlight therapeutic opportunities for side-specific CRC treatment, including hypoxia targeting in RCRC, *MMP7* ^+^ cancer cell targeting in advanced LCRC, and the potential disruption of immunosuppressive, angiogenic, and migratory axes.

Overall, research demonstrates that left-sided and right-sided colorectal cancer are fundamentally distinct diseases at the level of tumor cells. LCRC is characterized by a PS program, lower malignancy scores linked to *LPCAT2* ^+^ cells (which inhibit tumor growth and induce ferroptosis), enhanced mucosal healing/protein folding, and tumor cells localized in glycolytic niches interacting with specific immune cells (*PLTP* ^+^ macrophages, Tregs, *LAYN* ^*+*^ CD8^+^ cells). In contrast, RCRC is dominated by an IS program, exhibits pronounced glycolysis (especially in MAC), heightened inflammatory states, and tumor cells concentrated near hypoxic cores and interacting with distinct immune cells (*SPP1* ^+^ macrophages, *CD161* ^+^ CD8 T cells). Functionally unique subpopulations exist, including metastatic *MMP7* ^+^ cells in advanced LCRC and hypoxia-responsive subclusters in RCRC. These profound differences in molecular drivers, metabolism, tumor microenvironment, and cellular functions highlight the need for distinct, side-specific therapeutic strategies, such as targeting the LPCAT2/ferroptosis axis or *MMP7* ^+^ cells in LCRC, and glycolysis or hypoxia in RCRC.

#### 4.1.2 Mechanisms of immune cell signaling in LCRC and RCRC progression and immunotherapy

Sc-seq has revealed a complex and sometimes contradictory immunological landscape in CRC, where tumor sidedness is a major, although not exclusive, determinant of immune function. The collective findings on immune cells in regards to tumor side differences are compiled in Table 4.

The myeloid compartment demonstrates cellular and functional divergence in regards to tumor side. In LCRC, macrophages are frequently polarized toward an M1-like, pro-inflammatory state, exhibiting high expression of pro-inflammatory molecules and functional enrichment for inflammatory pathways, transcriptional regulation, and B-cell receptor signaling (Hu et al., 2025; Ji et al., 2024). Conversely, RCRC is dominated by M2-like, anti-inflammatory macrophages, which are defined by osteopontin (*SPP1*) expression and primarily interact with other leukocytes (Hu et al., 2025). These *SPP1* ^+^ macrophages in RCRC upregulate genes that regulate hypoxia, angiogenesis, and EMT, and their high infiltration is linked to a significantly shorter 5-year overall survival (OS) (Ji et al., 2024). However, these side-specific differences are nuanced by contradictory data. Firstly, the study of Hu et al. demonstrates that the accumulation of *SPP1* ^+^ macrophages during malignant progression is noted as a common feature in both LCRC and RCRC, suggesting their role is not entirely specific to the right side (Hu et al., 2025). Moreover, another study characterizes RCRC to exhibit a higher proportion of monocytes in comparison to LCRC (Shang et al., 2024), with these cells activating pathways involved in leukocyte proliferation, antigen presentation, and MHC binding - functions critical for pro-inflammatory macrophage activity. A key driver in this population is the lung cancer associated transcript 1-positive (*LUCAT1* ^*+*^) monocyte/macrophage subset, which sustains its own activation and contributes to the inflammatory TME by overexpressing the pro-tumorigenic CD74-CD44 axis (Shang et al., 2024; Shi et al., 2006).

Furthermore, the T cell compartment also demonstrates cellular and functional divergence based on tumor side. In LCRC this compartment is often indicative of a suppressed/dysfunctional state: LCRC T cells are predominantly naïve/undifferentiated and enriched with CD4^+^ T cells, with immune cells interacting mainly amongst themselves (Liu D et al., 2024). Furthermore, LCRC T cells display enrichment in stress-related signaling pathways, suggesting a state of chronic activation that may underly their poor response to immunotherapy (Hu et al., 2025). Conversely, the T cell landscape in RCRC is suggestive of a presumably higher immune activation state than in LCRC tumors. Here, the T cell population is enriched with highly differentiated and activated CD8^+^ T cells and engages in frequent and intense tumor-immune cell interactions. However, the nature of these interactions is complex: for instance, NECTIN2-TIGIT is shown to dampen immune response, whereas PLXNB2-SEMA4D presumably promotes immune cell activation and differentiation (Ch’ng and Kumanogoh, 2010; Deuss et al., 2017; Liu D et al., 2024). Moreover, the presence of exhausted T cells in this context may signify a prior active anti-tumor response, potentially predicting better response to therapy. Importantly, another finding highlights that the functional shift of CD8^+^ T cells from a cytotoxic to an exhausted state is actually a stage-dependent process that occurs regardless of the tumors side, suggesting that this may actually be a universal characteristic of CRC tumor biology irrespective of tumor origin (Hu et al., 2025).

The distribution and functional characteristics of B cells also vary based on the tumor site of origin. LCRC exhibits a near-complete depletion of naïve B cells and is characterized by immunosuppressive inter-immune B cell interactions, including immune cell activation-suppressing CD52-SIGLEC10 axis with myeloid cells (Bandala-Sanchez et al., 2018; Ji et al., 2024). These findings reinforce the pattern of a suppressed microenvironment consistent with observations in the myeloid and T cell niches. Conversely, B cells in RCRC play a more favorable role. Despite the majority of inter-immune communications with myeloid cells occurring in both sides (including pro-tumor pairs like SPP1-CD44, LAMP1-FAM3C, and MERTK-GAS6), *CD20* ^+^ B cells specific to the right side are a favorable prognostic marker and have been demonstrated to be essential for the efficacy of anti-PD-1 therapy in murine models and patient-derived organoids (Ji et al., 2024).

Although tumor origin site shapes the immune landscape according to several studies, some core features are conserved between LCRC and RCRC according to others. For instance, the overall expression of immune checkpoint ligands and receptors (ICLs/ICRs) show minimal variation, indicating that sidedness alone in this case is a poor predictor of response to checkpoint blockade (Liu et al., 2022). Furthermore, the pervasive inhibitory communication networks among myeloid, mast, B, and tumor cells via axes such as immune cell inhibition by tumor cells via CD24-SIGLEC10 and LGALS9-HAVCR2 interactions appear to be a fundamental characteristic of the CRC microenvironment, with minimal influence from sidedness (Barkal et al., 2019; Hill et al., 2025).

Overall, research reveals an immunological divergence between LCRC and RCRC that shapes their distinct TMEs, although discrepancies in its nature exist. On one hand, LCRC is described as immunosuppressive, with the T cell population enriched for naïve/undifferentiated CD4^+^ T cells and stress-related signaling pathways, suggesting that they are in a chronic activation state and, therefore, not effective for immunotherapeutic targeting. This is further supported by a nearly-complete depletion of naïve B cells, as well as enhanced immunosuppressive B cell-myeloid cell interactions (such as CD52-SIGLEC10). On the other hand, studies mention that macrophages in LCRC can exhibit high expression of pro-inflammatory molecules and show functional enrichment for inflammatory pathways [although it should be noted that this state of immune cell activation in LCRC may also suggest preceding of chronic inflammation (Zhao et al., 2021)]. Such differences likely stem from several methodological and biological variables. Firstly, cohort heterogeneity presents a significant challenge. In this case, differences in disease stage, prior treatments and molecular subtypes (such as microsatellite instability status) can significantly hinder site-specific comparisons. Secondly, technical divergences in analytical pipelines, such as variations in clustering resolution and cell type annotation criteria, as well as the principles for significant ligand-receptor interaction identification can lead to the characterization of fundamentally different cell populations/intercellular interactions across studies. Thirdly, spatial heterogeneity within the tumor itself due to sampling from either the tumor core or the invasive margin can capture distinct immunological niches, further complicating direct comparisons.

RCRC, in turn, is characterized as immunostimulatory in several studies, with enhanced leukocyte proliferation, antigen presentation and MHC binding in monocytes, as well as the T cell population being enriched for highly differentiated and activated CD8^+^ T cells. Moreover, RCRC shows frequent and intense immune-tumor cell interactions, including through enhancement of macrophage migration by the SEMA4A-PLXND1 axis or by pro-tumorigenic signaling between immune cells (*LUCAT1* ^+^ monocytes and macrophages) with the help of CD74-CD44, and possess metabolically active and highly dense *CD20* ^+^ B cells correlating with favorable prognosis and shown to be essential for anti-PD-1 therapy. Conversely, signs of immunosuppression have been noted, with macrophages in RCRC enriched for anti-inflammatory genes and primarily interacting with leukocytes. Such discrepancies may be described similarly as to those mentioned previously (both biological and methodological).

Importantly, several immune features show no consistent differences by tumor sidedness. The accumulation of exhausted CD8^+^ T cells and *SPP1* ^+^ macrophages during malignant progression was identified to occur in both LCRC and RCRC in several studies. Likewise, the core functional shift of CD8^+^ T cells from cytotoxicity to exhaustion was shown to be stage-dependent rather than location-dependent. CD4^+^ T cell subcluster enrichment in early/middle stages, along with overall patterns of ICL/ICR expression and broad cell-cell communication networks, show minimal variation by sidedness in some studies. *CD20* ^+^ B cells are strongly linked to improved prognosis and enhanced response to anti-PD-1 therapy in RCRC, but B-cell interactions with myeloid cells occur in both tumor locations.

Thus, these findings suggest that, although not unanimously, tumor sidedness informs, but does not exclusively dictate, immunotherapy strategies. One of the options to further clarify the aforementioned findings is for future studies to adopt standardized processing and analysis protocols, coupled with more refined patient stratification that accounts for the multifaceted nature of the disease. Currently, it can be proposed that LCRC may benefit from interventions that convert chronic inflammation into productive antitumor immunity (for example, enhancing antigen presentation or disrupting Treg suppression). RCRC, on the other hand, is immunostimulatory, and enhancing immune signaling crosstalk, such as SEMA4A-PLXND1 between immune and tumor cells could be advantageous. Importantly, both tumor types harbor macrophages with adverse functions that contribute to both immunostimulation and immunosuppression. Therefore, further research is critical to understand the signaling mechanisms of these distinct macrophage populations.

#### 4.1.3 Mechanisms of stromal cell signaling in LCRC and RCRC progression

A critical emerging theme in CRC biology is that tumor sidedness dictates fibroblast function and stromal remodeling (Ahmad Zawawi and Musa, 2022) (Table 5).

LCRC stroma is defined by heterogeneity presented by a specific pro-tumor CAF subset. A sc-seq study revealed that CAFs express high levels of phosphate regulon transcriptional regulatory protein B (*RHOB*) to promote cell growth and migration, and a distinct myosin heavy chain 11-positive (*MYH11* ^+^) CAFs population is highlighted (C. Wang et al., 2024). This subset ultimately acts as a regulator of LCRC aggressiveness, promoting tumor invasion through TGF-β signaling, upregulation of pro-tumor genes (*RAMP1, MYLK, MYH11*) and activation of muscle-invasive pathways. Apart from tumor invasion promotion, these CAFs potentially form an immunosuppressive niche through inhibition of immune cell migratory (myeloid cells) CD74-MIF and SPP1-CD44 ligand-receptor signaling interactions. These processes together drive tumor progression and highlight *MYH11* ^+^ CAFs as a potential therapeutic target in LCRC.

In RCRC, CAFs also promote tumor progression but primarily function as drivers of angiogenesis in addition to invasion (Hu et al., 2025). They express proteins like AGR2 to potentiate VEGF and FGF2 signaling, and REG4 to intensify tumor invasion. This process is driven by transcription factors like *E2F1* and *FOXF1* which promote rapid tumor growth by activating cell cycle and apoptotic pathways that contribute to neovascularization.

Beyond the location-specific stroma signaling, universal mechanisms of CAF activity have been noted (Hu et al., 2025). Across all CRC subtypes, CAFs tend to engage in pro-tumor functions with pathways like “focal adhesion” and “ECM-receptor interaction” being activated. Moreover, a distinguished *Stromal3* ^+^ fibroblast subset is utilized by tumors for building of the ECM. Starting from the earliest stages, CAFs across the colorectum exhibit WNT signaling - a key pathway for invasion promotion - marked by specific regulation of the transcription factor TCF4.

Thus, comparative analyses underscore the impact of tumor sidedness on CAF function. RCRC CAFs promote angiogenesis (via *AGR2*) and invasion (via *REG4* and transcription factors *E2F1/FOXF1*), whereas LCRC CAFs are characterized by greater heterogeneity and enrichment of *MYH11* ^+^ CAFs, which promote invasion, immunosuppression (via CD74-MIF/SPP1-CD44), and metastasis. Despite these differences, CAFs across both CRC subtypes are shown to activate ECM-remodeling and WNT pathways, helping the tumor to progress. Overall, in addition to the general molecular signaling pathways activated in CAFs to facilitate the progression of the tumor, the anatomic origin of CRC tumors defines distinct stromal pro-tumor programs, making location-specific CAF subsets and their signaling pathways potential therapeutic targets.

## 5 Conclusion and perspectives

Recent sc-seq and spatial transcriptomic studies have provided insight into CRC progression by describing it as a complex ecosystem of tumor, immune, and stromal cells. The application of sc-seq and spatial transcriptomic technologies has shed light onto CRC progression, therapy resistance and metastasis mechanisms. This review synthesizes how these methods have shifted our understanding of tumor biology, revealing CRC as a complex, sometimes functionally heterogenous ecosystem combining tumor, immune, and stromal cells. Importantly, this ecosystem is not uniform. Its structure, functions, and response to therapy are dependent on the tumor’s embryological origin - left-sided or right-sided. The research on CRC tumor sidedness signaling differences help to understand CRC biology and therefore design more effective therapeutic strategies.

Importantly, a major challenge in interpreting and comparing the studies discussed in this review is their discrepant findings, particularly regarding immune signaling mechanisms. Such inconsistencies may arise due to both methodological and biological variables. Key factors include heterogeneity in patient cohorts such as the disease stage, prior treatment administration and the tumor molecular subtypes like the microsatellite instability status. Furthermore, sampling from different tumor regions (e.g., the tumor core versus the margin) can capture distinct cellular niches, complicating direct comparisons across studies. Technical variations in data analysis further contributes to divergent results. Differences in clustering resolution, cell type annotation, and criteria for identifying significant ligand-receptor interactions can lead to the characterization of fundamentally different cell populations and communication networks. This methodological variability underscores the need for future studies to adopt standardized workflows for single-cell data processing and analysis coupled with more refined patient stratification that accounts for the complex nature of the disease. It also highlights the necessity for biological validation of computational findings both *in vitro* and *in vivo*. Despite these challenges, the co-signaling between cancer cells and the TME remains a critical area for further research, as new mechanisms are continually being uncovered.

Single-cell analyses have revealed that tumor progression, therapy resistance, and metastasis in CRC arise largely from dynamic cellular crosstalk between tumor cells and the TME. Several mechanisms underpinning these processes can be highlighted in overall CRC. Firstly, cellular plasticity within the primary tumor contributes to divergent functional outcomes. For instance, pro-tumorigenic *FAP* ^+^/*SPP1* ^+^/*CTHRC1* ^+^ CAFs drive immunosuppression and invasion whereas immunogenic *PI16* ^+^/*SLIT2* ^+^ CAFs promote T-cell recruitment and activation. Similarly, macrophages can be *C1QC* ^+^ and immunostimulatory or *SPP1* ^+^/*SIRPA* ^+^ and immunosuppressive (Table 4). Secondly, the spatial organization of cells within the primary tumor mass determines their function. For example, pMMR tumors form organized and immune-excluding barriers, while dMMR tumors exert proximity-dependent interactions (e.g., predicted *CXCL13* ^+^ T cells-*LAMP3* ^+^ DCs interactions via PD-1/PD-L1) that determine immunotherapy response. Third, metabolic and signaling focal points drive cell coordination. NU^high^ tumor cells, regulated by *NME1*, act as signaling hubs, interacting with stromal and immune cells via ligands like immune-suppressing MIF or immune cell activating PLA2 (Table 2). Moreover, the pro-tumorigenic KITLG-KIT signaling axis between stromal and immune cell compartments defines an anti-tumor niche, while the VEGFA-VEGFR-ESM1 positive feedback loop in endothelial tip cells promotes angiogenesis and immune evasion (Table 5). In metastases, cancer cells undergo a shift toward immune silence and reduced intercellular interactions, as well as reprogramming of macrophages into immunosuppressive *SPP1* ^*+*^ *, MRC1* ^*+*^ *CCL18* ^*+*^ *,* and *THBS1* ^+^ phenotypes. NAC further remodels this immune landscape, promoting a transition of TAMs from activated to immunosuppressive states. Lastly, *SFRP2* ^+^ CAFs are demonstrated to promote tumor growth and metastasis. Together, these findings underscore key cellular interactions and specific cell subtypes as potential new therapeutic targets for both overall primary and metastatic CRC.

Importantly, the two major CRC subtypes - LCRC and RCRC - are not solely anatomically distinct tumors but functionally divergent diseases with distinguishable cellular ecosystems (Tables 2–5). This may explain their different clinical behaviors and responses to treatment. LCRC is characterized as a tumor subtype featuring *MMP7* ^+^ matrix remodeling, PS meta-program characterized by expression of genes associated with proliferation and stemness, elevated expression of *PHLDA2* linked to lymph node metastasis and minimal MHC I expression in tumor cells, while harboring an *LPCAT2* ^+^ tumor cell population that decreases cell proliferation. The immune cell population, in turn, is characterized as immunosuppressive in a number of studies, with the T cell population enriched for naïve/undifferentiated and exhausted CD4^+^ and CD8^+^ T cells and stress-related signaling pathways, suggesting that they are in a chronic activation state. These findings are further supported by the absence of naïve B cells. On the other hand, other studies mention that macrophages in LCRC can exhibit high expression of pro-inflammatory molecules and show functional enrichment for inflammatory pathways. Additionally, a population of *MYH11* ^+^ CAFs expresses pro-tumor and migration genes. RCRC tumor ecosystem, in turn, is comprised of tumor cells with high MHC I expression, characterized by a IS meta program and increased hypoxia in comparison with LCRC. Furthermore, immune cells are characterized as immunostimulatory in several studies, with T cells present in a recently active state and early stage CD8^+^ T cells, *LUCAT1* ^+^ monocytes expressing genes related to interleukin production, as well as *CD20* ^+^ B cells correlating with a favorable prognosis. Contrarily, sings of immunosuppression have been identified in other studies, with the presence of *SPP1* ^+^ macrophages enriched for anti-inflammatory genes and a *CD161* ^+^ T cell subset correlating with lower OS being detected. These discrepancies are further complicated by another group of studies that demonstrate that several immune features show no consistent differences by tumor sidedness, for example, the accumulation of exhausted CD8^+^ T cells and *SPP1* ^+^ macrophages during malignant progression was identified to occur in both LCRC and RCRC. Lastly, CAFs in RCRC were identified to express angiogenesis- and tumor growth-promoting *AGR2* and *REG4* genes. These findings point to the complexity of tumorigenic signaling within overall CRC and its subtypes, making it troubling to target their cell-specific signaling pathways.

Cell-cell communication analysis examines how the binding of ligands to receptors influences cellular behavior and fate. This analytical approach, widely applied in sc-seq and spatial transcriptomics data analysis using tools such as CellChat, CellPhoneDB, allows to understand the diverse cellular decisions, including decisions to activate the cell cycle or apoptosis, undergo migration or differentiate along the lineage (Efremova et al., 2020; Jin et al., 2021). In CRC, extensive crosstalk has been uncovered between tumor cells and the TME in overall and sided CRC (Table 2). In overall CRC, a significant portion of the cell-cell crosstalk involves tumor cells communicating with fibroblasts and endothelial cells. Some of these interactions include: 1) integrin-mediated signaling—interactions of collagens (COL1A1, COL4A1) and laminins with integrin receptors (ITGB1, ITGAV) are a universal mechanism for enhancing tumor cell migration, invasion, and adhesion; 2) Wnt and Hedgehog pathways—signaling through IHH-PTCH1 and WNT5A-FZD/LRP participates in the regulation of stromal cell differentiation and creating an immune-restrictive ECM; 3) regulation of angiogenesis—the VEGFA-NRP1/2 axis participates in new blood and lymphatic vessels formation. Moreover, the TME actively suppresses anti-tumor immunity through several ligand–receptor–mediated mechanisms, including: 1) checkpoint inhibition by B cell-T cell interactions via the PD-L1/PD-1 axis; 2) CD47-SIRPA and CD52-SIGLEC10 interactions protecting tumor cells from phagocytosis by macrophages; 3) signals from stromal and tumor cells (such as LGALS9-HAVCR2 and CSF1-CSF1R) promoting pro-tumor, M2-like macrophage polarization; 4) a balance of co-stimulatory (CD28^−^CD80/86) and co-inhibitory (CTLA4-CD80/86) signals regulating the T-cell response.

Moreover, cell-cell communication analysis revealed distinct biological preferences between the left and right sides of CRC (Table 2; Figure 1). LCRC is characterized by a more reactive tumor stroma and a growth factor-driven TME: ligand-receptor interactions are shifted towards a tumor-stromal crosstalk, particularly with fibroblasts. Pathways like PLAU-PLAUR and TGFB1-TGFBR1 drive the formation of inflammatory CAFs, collagen deposition, and direct tumor cell proliferation. Furthermore, a notable feature for LCRC is the recruitment and instruction of immunosuppressive B cells via the chemokine-mediated CCL28-CCR10 axis. In contrast, RCRC has an immune cell-signaling TME, dominated by chemokine signaling (e.g., CCL15-CCR1 and CXCL16-CXCR6) that actively recruits specific immunosuppressive *SPP1* ^+^ macrophages yet PD-L1 interacting *CD161* ^+^ CD8^+^ T cells. These recruited cells then participate in immunosuppressive signaling: *SPP1* ^+^ macrophages dampen T-cell cytotoxicity via SPP1-CD44, while tumor cells directly inhibit T cell function by PD-L1-CD80 and TIGIT-NECTIN2 signaling. Lastly, RCRC utilizes signaling pathways through ligand-receptor pairs like DCN-VEGFR2 and SEMA3B-NRP1/2 to promote metastasis and vascular permeability, differing from the canonical VEGFA-driven angiogenesis. Collectively, these findings highlight that while both overall and sided CRC rely on core signaling pathways for the promotion of tumor growth and invasion, the specific ligand-receptor interactions underpinning these processes are sidedness-dependent. LCRC is driven by a stromal and growth-factor-oriented TME, whereas RCRC is characterized by an inflammatory yet immunosuppressive ecosystem. There distinctions underscore the need for subtype-specific therapeutic strategies. We propose that the enhancement of CRC therapy efficiency potentially lies in targeting the aforementioned cell-cell interactions. Such intervention could (i) overcome therapy resistance, by disrupting protective tumor-TME-signaling networks, and (ii) prevent immune cell therapy evasion, by blocking immunosuppressive ligand-receptor interactions that inhibit anti-tumor immune responses.

In order to translate the aforementioned findings into clinical progress, future research should aim to prioritize the adoption of single-cell multi-omics technologies. While sc-seq captures a cell’s phenotypic/transcriptional state, it provides a limited view of the cells’ molecular networks controlling cell identity and function. Single-cell multi-omics methods which simultaneously capture genomic, epigenomic, transcriptomic and proteomic networks in the same cells are essential to overcome this limitation (Bai and Zhu, 2023). This integrated approach can directly explore key biological questions. For instance, methods like G&T-seq profile the genome variation and transcriptome of cells simultaneously and can be utilized to assess the origin and evolution of tumors from LCRC/RCRC with enhanced sensitivity. Furthermore, the established link between RCRC and the CpG island methylation phenotype remains mechanistically unclear (Lee et al., 2017). Applying methods that co-profile the methylome and transcriptome (such as snmCAT-seq for base-resolution cytosine methylation or scATAC-seq to map chromatin accessibility) can directly connect epigenetic landscapes to the gene expression programs that drive sidedness. Furthermore, because transcript levels often poorly predict protein abundance, methods integrating the transcriptome with proteome (for example, CITE-seq/REAP-seq) are critical to determine how the distinct transcriptional networks of LCRC and RCRC are functionally translated into protein-level effectors, revealing the functional mechanisms of tumor divergence. A limitation of the aforementioned methods is the loss of spatial architecture analysis, which is fundamental for understanding tumor signaling. While the studies compiled in our review have begun to map predicted cell-cell interactions at the RNA level, the next step is to integrate spatial context with multi-omics technologies. Emerging spatially resolved multi-omics technologies, for instance, spatial-ATAC-RNA-seq use deterministic barcoding to co-profile accessible chromatin or histone modifications with the transcriptome on the same tissue section. These methods are poised to uncover new biological insights into region-specific epigenetic priming and gene regulation within the distinct histological landscapes of LCRC and RCRC.

The findings summarized in this review may help guide future clinical trial design by moving toward more targeted approaches that account for the complex ecosystem of LCRC and RCRC. The development of future trials aiming to stratify patients based on integrated “TME subtypes” that incorporate tumor sidedness with specific cellular and molecular features may hold promise for increasing the survival of CRC patients. For instance, for LCRC patients whose tumors are characterized by a reactive stroma enriched in *MYH11* ^+^ CAFs and prominent TGF-β signaling, a potential combination therapy could pair immune checkpoint inhibition with TGF-β pathway inhibitors or specific anti-stromal agents. Moreover, for RCRC patients with an *SPP1* ^+^ macrophage-rich niche and evidence of *CD161* ^+^ T cell-mediated immunosuppression, trial designs could combine immune checkpoint inhibition with agents targeting the SPP1-CD44 axis or other macrophage-reprogramming drugs. Implementing such a precision approach will require the simultaneous development of auxiliary diagnostic assays to reliably identify these TME subtypes in patient biopsies, and may potentially be derived from spatial transcriptomics and multi-omics data. Overall, the combined utilization of specific TME features with corresponding combination therapies hold promise for increasing the likelihood of clinical success and overcoming of therapy resistance in left- and right-sided CRC.

In summary, single-cell technologies have given insight into overall and sided CRC cellular structure. These findings allow moving beyond treatments based solely on the location of the tumor, but instead focus on designing therapies that target the entire network of cells within the tumor. This shift is crucial for overcoming tumor progression, therapy resistance and metastasis in CRC.

## Glossary

5-FU: 5-fluorouracil
BAFF: B cell-activating factor
BHLHE40: basic helix-loop-helix protein 40
BTK: Bruton tyrosine kinase
C1QC: C-chain polypeptide of serum complement subcomponent C1q
CAFs: cancer-associated fibroblasts
CCL8: C-C motif chemokine ligand 8
CD103: integrin αE
CD38: cyclic ADP ribose hydrolase
CR: complete response
CRC: colorectal cancer
CSF1R: colony stimulating factor 1 receptor
CTHRC1: collagen triple helix repeat containing 1
CXCL1: growth-regulated oncogene-α*-positive*
CXCL12: stromal derived factor 1
CXCL13: chemokine ligand 13
CXCR4: chemokine receptor type 4
DC: dendritic cell
DCN: decorin
dMMR: deficient MMR
ECM: extracellular matrix
ECs: endothelial cells
EMT: epithelial-mesenchymal transition
F4/80: adhesion G protein-coupled receptor
FAP: fibroblast activation protein α
GNLY: granulysin
*GPX4*: glutathione peroxidase 4
GSEA: gene set enrichment analysis
GSVA: gene set variation analysis
HDAC: histone deacetylase
ICL: immune checkpoint ligand
ICR: immune checkpoint receptor
IHC: immunohistochemical
IS meta-program: immune secretory meta-program
IS: immune secretory
ITAMs: immunoreceptor tyrosine-based activation motifs
KO: knockout
LAG3: Lymphocyte-Activation Gene 3
LAMP3: lysosomal-associated membrane protein 3
LAMs: Lipid-associated macrophages
LCRC: left-sided colorectal cancer
LPCAT2: lysophosphatidylcholine acyltransferase 2
LpMs: lamina propria macrophages
LYVE1: lymphatic vessel endothelial hyaluronan receptor 1-positive
MAC: mucinous adenocarcinoma
mCRC: metastatic colorectal cancer
MCs: mast cells
MIF: macrophage migration inhibitory factor
MMPs: matrix metalloproteinases
MMR: mismatch repair
MMs: muscularis macrophages
MSLN: mesothelin
MTRGs: membrane-tension related genes
MYH11: myosin heavy chain 11
NAC: neoadjuvant chemotherapy
NME1: nucleoside diphosphate kinase A
NR: non-responders
**NU** ^**high**^ **cells**: high nucleotide metabolism cells
OS: overall survival
PD-1: programmed cell death protein 1
PHLDA2: pleckstrin homology like domain family A member 2
PI16: peptidase inhibitor 16
PLA2: secreted phospholipase A2
pMMR: proficient MMR
PMN: pre-metastatic niche
PPAR: peroxisome proliferator-activated receptor
PR: partial response
PS meta-program: proliferation stemness meta-program
PS: proliferation stemness
RCRC: right-sided colorectal cancer
RHOB: phosphate regulon transcriptional regulatory protein **B**
Sc-seq: single-cell sequencing
SD: disease stabilization
SFRP2: frizzled-related protein 2
SIRPA: signal regulatory protein α
SLIT2: slit guidance ligand 2
SmMs: submucosal macrophages
SPP1: osteopontin
SYK: spleen tyrosine kinase
TAMs: tumor-associated macrophages
Tfh: T follicular helper
TFRC: transferrin receptor
TIMP1: tissue inhibitor of metalloproteinase 1
TME: tumor microenvironment
TMSB4X: thymosin beta-4
